# Improving postsurgical paresis in brain tumor patients by transcranial magnetic stimulation

**DOI:** 10.1007/s11060-024-04931-9

**Published:** 2025-01-23

**Authors:** Tizian Rosenstock, Thomas Picht, Melina Engelhardt, Ulrike Grittner, Maximilian Mönch, Peter Vajkoczy, José Pedro Lavrador, Ana Mirallave-Pescador, Francesco Vergani, Maximilian Schwendner, Axel Schroeder, Leonie Kram, Haosu Zhang, Sujit Prabhu, Sarah Prinsloo, Bernhard Meyer, Sebastian Ille, Sandro M. Krieg

**Affiliations:** 1 Department of Neurosurgery, Charité – Universitätsmedizin Berlin, Corporate Member of Freie Universität Berlin, Humboldt-Universität zu Berlin, Berlin Institute of Health, Charitéplatz 1, 10117 Berlin, Germany; 2 BIH Charité Digital Clinician Scientist Program, Berlin Institute of Health at Charité – Universitätsmedizin Berlin, BIH Biomedical Innovation Academy, Charitéplatz 1, 10117 Berlin, Germany; 3 Cluster of Excellence: “Matters of Activity. Image Space Material, ” Humboldt University, Unter den Linden 6, 10099 Berlin, Germany; 4 Einstein Center for Neurosciences, Charité – Universitätsmedizin Berlin, Corporate Member of Freie Universität Berlin, Humboldt-Universität zu Berlin, Berlin Institute of Health, Charitéplatz 1, 10117 Berlin, Germany; 5 Institute of Biometry and Clinical Epidemiology, Charité – Universitätsmedizin Berlin, Corporate Member of Freie Universität Berlin, Humboldt-Universität zu Berlin, Berlin Institute of Health, Charitéplatz 1, 10117 Berlin, Germany; 6 Neurosurgical Department, King’s College Hospital Foundation Trust, London, UK; 7 Neurophysiology Department, King’s College Hospital Foundation Trust, London, UK; 8 Department of Neurosurgery, School of Medicine, Technical University of Munich, Klinikum rechts der Isar, Munich, Germany; 9 Department of Neurosurgery, Heidelberg University Hospital, Ruprecht-Karls-University, Im Neuenheimer Feld 400, 69120 Heidelberg, Germany; 10 Department of Neurosurgery, The University of Texas MD Anderson Cancer Center, Houston, TX USA

**Keywords:** Glioma, Paresis, Navigated transcranial magnetic stimulation (nTMS)

## Abstract

**Background and objectives:**

Recently, reduction of transcallosal inhibition by contralateral navigated repetitive transcranial magnetic stimulation (nrTMS) improved neurorehabilitation of glioma patients with new postoperative paresis. This multicentric study examines the effect of postoperative nrTMS in brain tumor patients to treat surgery-related upper extremity paresis.

**Methods:**

This is a secondary analysis of two randomized and three one-arm studies in brain tumor patients with new/progressive postoperative paresis. Patients underwent either low frequency contralesional nrTMS or sham stimulation followed by physiotherapy. Outcome was assessed on postoperative day 1, 7, and after 3 months using British Medical Research Council score (BMRC), Fugl-Meyer assessment (FMA), Karnofsky Performance Scale (KPS) and National Institutes of Health Stroke Scale (NIHSS).

**Results:**

A total of 135 patients (mean age of 53.8 years, 60 women) were included, of whom 51 patients were treated in RCTs (30 treatment group, 21 sham group) and 84 in prospective, single-arm studies. Linear mixed models showed an advantage for the treatment group for the BMRC (7 days: OR 3.28; 95%CI: 1.08–9.99; 3 months: OR 2.03, 95%CI: 0.65–6.39) and KPS (7 days: mean difference (MD) 11, 95%CI: 2–19; 3 months: MD 11, 95%CI: 2–20), less pronounced for the FMA (7 days: MD 0.28, 95%CI: -0.34-0.9; 3 months: MD 0.14, 95%CI: -0.52-0.81). A stronger treatment effect was evident with proven ischemia on the postoperative MRI. To observe an improvement by at least one grade at 3 months, the number needed to treat (NNT) for the entire cohort is 4 (BMRC) and 3 patients (KPS), respectively.

**Conclusion:**

Our multicenter data confirm the positive treatment effect of nrTMS to reduce transcallosal inhibition with a considerably low NNT - especially if caused by ischemia.

**Supplementary Information:**

The online version contains supplementary material available at 10.1007/s11060-024-04931-9.

## Introduction

In the endeavor of reaching the maximum extent of resection, surgery for brain tumors may cause significant functional deficits despite the use of intraoperative neuromonitoring technologies. Especially for tumors located in or close to the pyramidal tract, surgery-related paresis can be a severely impeding sequelae, reducing quality of life and eligibility for adjuvant therapy. In the majority of cases, postoperative paresis originates from subcortical ischemia rather than resection of eloquent brain tissue [1]. Due to reduced mobility and independence with consecutive ineligibility for adjuvant therapy, patients’ overall survival is further reduced [2]. A new postoperative deficit has such a significant negative impact on survival that the apparent advantage of gross-total / supramarginal resection is negated [3].

Navigated repetitive transcranial magnetic stimulation (nrTMS) showed variable effectiveness for therapy of stroke patients in several randomized studies, depending on the size and cause of their ischemic lesions [4]. Especially for patients with large lesions, chances of functional reorganization and thus recovery were small. These patients showed various ipsilesional mechanisms promoting functional recovery from ischemic tissue loss with resulting disability [5, 6]. The contralesional hemisphere influences ipsilesional functional improvement by transcallosal inhibition (TCI) [7, 8]. Under healthy conditions, upper extremity (UE) movement is coordinated by both hemispheres via motor cortex interaction, enabling uni- and bi-manual and preventing mirror movements [9]. After unilateral injury, such as stroke, TCI can worsen functional recovery since the healthy hemisphere inhibits the contralateral ischemic motor system, thus limiting recovery [10, 11]. Non-invasive approaches to reduce TCI originating from the contralesional motor cortex have been developed [12]. Low-frequency repetitive transcranial magnetic stimulation (rTMS) reduced the motor resting threshold of the patients and increased the motor-evoked potential of the non-stimulated motor cortex (affected motor cortex), while opposite effects were observed in the stimulated motor cortex (non-affected motor cortex) [13].

nrTMS is a modern tool to non-invasively modulate brain networks. Given its growing availability across centers and its capacity to precisely stimulate the motor cortex, nrTMS is particularly well-suited for modulating cortical excitability, especially in brain tumor patients. Specifically, low-frequency nrTMS increases contralateral cortical excitability by reducing excitability of the ipsilateral motor system [14, 15]. In this application of contralesional motor cortex inhibition after stroke, rTMS demonstrated its effectiveness in enhancing motor function, albeit with varying success rates across several studies [16–19]. Two recently published RCTs on glioma patients with surgery-related subcortical ischemia demonstrated excellent recovery and a unexpectedly strong effect of contralesional nrTMS in the acute phase during the first postoperative week [20, 21]. 

The present multicentric study therefore examines the effect of postoperative nrTMS of the unaffected hemisphere in brain tumor patients to treat surgery-related upper extremity paresis on the first days after surgery combined with physical therapy (PT).

## Methods

### Eligibility criteria

Patients above 16 years developing surgery-related paresis (= worse British Medical Research Council (BMRC) score than preoperative) of the UE immediately after brain tumor resection were enrolled. The center-specific inclusion criteria are presented in Table 1. Exclusion criteria were usual nrTMS and MRI exclusion criteria (pacemaker, brain electrodes, and cochlear implants) [22]. All patients were operated using motor-evoked potentials and subcortical stimulation according to previously published standards [23]. Although awake craniotomies were not an exclusion criterion per se, all patients were operated on while asleep. Biopsy only cases were not enrolled.

**Table 1:** Stimulation setup

	center	I	II	III	IV
**Enrollment**	subcortical ischemia*	yes	yes	yes	no
	SMA tumors included	yes	no	yes	yes
	MEP present#	**not assessed**	yes	**not decisive for enrolment**	yes
**nrTMS**	Frequency (Hz)	1	1	1	1
	% RMT	110%	110%	110%	110%
	Location	contralesional M1, FDI hotspot	contralesional M1, FDI hotspot	contralesional M1, FDI hotspot	contralesional M1, FDI hotspot
	Pulses per session	900	900	900	2,000
	Number of days	7 (weekdays only)	7 consecutive	7 consecutive (or less if patients recover to MRC 5 before 7 days)	10 consecutive
**Physiotherapy**	During nrTMS	no	no	yes	no
	Within 1 h after nrTMS	yes	yes	No but daily PT on the ward	yes
	Duration (min)	30	30	30 min	20

This table provides an overview on the stimulation protocols of all 4 centers (I-IV). RMT = resting motor threshold; SMA = supplementary motor area. *new subcortical ischemia within the CST (as shown by diffusion-weighted imaging [DWI] in postoperative magnetic resonance imaging [MRI] scan) present in all cases. # Patients without preserved motor evoked potential (MEP) responses as measured by postoperative navigated TMS (nTMS) motor mapping were not included

### Study protocol

Patients underwent a navigational cranial MRI scan on the first postoperative day. Then, patients received low-frequency nrTMS treatment to the contralesional hemisphere’s hand motor hot spot according to center-specific protocols for 7–10 days (Table 1). All centers used an electric-field navigated TMS device to accurately calculate the induced electric field strength and location (Nexstim eXimia 4.3 or 5.1; Nexstim Plc, Helsinki, Finland). To apply nrTMS to the contralesional hemisphere’s motor hot spot, the stimulating coil was perpendicularly aligned to the gyrus according to the orientation of central sulcus pyramidal cells. For the RCTs, the sham stimulation was ensured using a plastic adapter, so that the sensations on the head and the sounds were the same (Center I). In contrast, Center II employed an extremely low stimulation intensity (5% RMT). For both cases, no relevant electromagnetic induction occurred in the cortex. Patients received intensive task-oriented PT for UE even during stimulation or scheduled ≤ 1 h.

### Outcome measures

In this secondary analysis all outcomes are analyzed exploratory. Functional motor status was assessed on postoperative day 1, at 7 days, and at 3-month follow-up using BMRC score and Fugl-Meyer assessment (FMA) of the UE [24, 25]. In addition, the Karnofsky Performance Scale (KPS) and National Institutes of Health Stroke Scale (NIHSS) were collected to evaluate (in-)dependence and neurological impairment in daily living. Since tumor progression can be a substantial confounder, functional status was not assessed later than 3 months after surgery [26, 27]. 

### Statistical analysis

The analysis was initiated retrospectively and performed using SPSS Statistics (29.0; IBM) and R 4.2.2 (R Foundation for Statistical Computing, Vienna, Austria). The data from the different centers are heterogeneous and the inclusion criteria vary slightly. In order to adequately address these limitations, different methods were used to enable valid analyses: outcome group differences were tested by linear mixed models (log-transformed FMA, KPS, NIHSS) and generalized linear mixed models (ordinal model, BMRC) with random intercept (patients), fixed effects (group allocation, study, time point, interaction term for timepoint / group and group / center, tumor histology, tumor location, presence of supplementary motor area (SMA) tumors and the particular baseline value). Mean differences and confidence intervals for the log-transformed FMA Score are reported on the log scale and for the NIHSS and KPS scores on the original data scale. The intervention effect of the BMRC score is reported as odds ratios to achieve a higher BMRC value for treatment versus sham.

Subgroup analyses were conducted for the following patient groups: those with motor-eloquent ischemia detected on postoperative MRI within the course of the corticospinal tract (CST) (*n* = 79), those without surgery in the SMA area (*n* = 94), those who received adjuvant radiotherapy (*n* = 69), and those with glioma tumor histology (*n* = 102). Other intended subgroup analyses (e.g. with maintained postoperative motor-evoked potentials (MEPs) or the cingulate gyrus) could not be performed due to small sample sizes.

To calculate the number needed to treat (NNT) for achieving a better long-term outcome (3 months after surgery), a binary logistic mixed model was utilized. The model included a random intercept for patients, fixed effects for baseline value, group allocation, time point, and an interaction term for time point/group.

Complete case analysis was done only as sensitivity analyses without imputation of informative or non-informative missings. Informative missings of outcome values were imputed with worst possible value if the patient died (*n* = 4 patients, FMA, KPS and BMRC = 0, NIHSS = 24), had a recurrence or reduced health status (*n* = 6 patients, FMA = 20, KPS, BMRC = 0, NIHSS = 24) or imputed with best possible outcome if fully recovered (*n* = 2 patients, FMA = 66, KPS = 100, BMRC = 5, NIHSS = 0). After single imputation of informative missings, remaining missing values were imputed using multiple imputation by chained equations assuming missing at random or missing completely at random yielding 30 complete data sets. Variables used for the estimation of multiple imputed data were: treatment group, center, age / sex of the patients, KPS, NIHSS, FMA, time point of measure, extent of resection, complication during surgery, tumor histology, tumor side, tumor location, use of antiepileptic drugs, MEPs maintained postoperatively, resting motor threshold (RMT)_tumor hemisphere_, RMT_healthy hemisphere_, ischemia, interaction of treatment group and time point of measure. Following guidelines on missing data in confirmatory clinical trials, outcome analyses based on imputed data are more reliable than the complete case analysis, which should be interpreted cautiously and is therefore only reported as sensitivity analysis.

### Data Availability

Data will be made available upon reasonable request after individual review of the request by the four involved ethics committees.

## Results

### Baseline characteristics

Between June 2015 and January 2023, 135 patients (60 females; 44.4%) were enrolled in the 4 centers, with 114 patients (84.4%) receiving nrTMS treatment according to the protocols shown in Table 1. As part of local RCTs, 30 patients (22.2%) were allocated to the treatment group and 21 patients (15.6%) to the sham group. The patients’ baseline characteristics are shown in Table 2.

**Table 2:** Patients’ characteristics by group assignment

		Group assignment		Absolute SMD
	total *n* = 135	rTMS stimulation *n* = 114	Sham stimulation *n* = 21	
**Study center**				
I^RCT^	30 (22.2%)	15 (13.2%)	15 (71.4%)	
II^RCT^ II^a^	21 (15.6%) 40 (29.6%)	15 (13.2%) 40 (35.1%)	6 (28.6%) -	
III	26 (19.3%)	26 (22.8%)	-	
IV	18 (13.3%)	18 (15.8%)	-	
**Age in years**, mean (SD)	53.8 (14.0)	53.4 (14.0)	56.1 (14.2)	0.20
**Sex** (female)	60 (44.4%)	52 (45.6%)	8 (38.1%)	0.15
**Hemisphere** (right)	59 (43.7%)	50 (43.9%)	9 (42.9%)	0.24
**Lobe**				
frontal	52 (38.5%)	46 (40.4%)	6 (28.6%)	0.72
parietal	19 (14.1%)	18 (15.8%)	1 (4.8%)	
temporal	10 (7.4%)	8 (7.0%)	2 (9.5%)	
insula	19 (14.1%)	12 (10.5%)	7 (33.3%)	
cingulate gyrus	8 (5.9%)	6 (5.3%)	2 (9.5%)	
multilocular	20 (14.8%)	18 (15.8%)	2 (9.5%)	
other	7 (5.2%)	6 (5.3%)	1 (4.8%)	
**Histology**				
glioma WHO grade 1	1 (0.7%)	1 (0.9%)	0	0.63
glioma WHO grade 2	7 (5.2%)	6 (5.3%)	1 (4.8%)	
glioma WHO grade 3	28 (20.7%)	23 (20.2%)	5 (23.8%)	
glioma WHO grade 4	66 (48.9%)	53 (46.5%)	13 (61.9%)	
metastases	16 (11.9%)	16 (14.0%)	0	
other	17 (12.6%)	15 (13.2%)	2 (9.5%)	
**Recurrent tumor**	18 missing 33 (28.2%)	18 missings 29 (30.2%)	4 (19.0%)	0.26
**EOR** (GTR)	13 missings 88 (72.1%)	11 missings 75 (72.8%)	2 missings 13 (68.4%)	0.10
**Ischemia**	20 missings 79 (68.7%)	62 (65.3%)	17 (85.0%)	0.47
**MEPs preserved postop.**	46 missings 55 (61.8%)	32 missings 48 (58.5%)	14 missings 7 (100%)	1.20
**Surgery within SMA area**	18 missings 23 (19.7%)	18 missings 22 (22.9%)	1 (4.8%)	0.54
**Postoperative radiotherapy**	21 missings 69 (60.5%)	20 missings 53 (56.4%)	1 missing 16 (80.0%)	0.52

The table shows characteristics of all 4 centers (I-IV)

SMD: standardized mean difference, standardized effect size which is Cohen’s d in case of continuous measures with normal distribution and extensions to multinomial and binary variables as proposed by Austin [34] and Yang & Dalton [35]

In the sham group, the proportion of insular tumors was higher (33.3% vs. 10.5%), which accounts for the higher proportion of motor-eloquent ischemia (85.0% vs. 65.3%). In addition, tumors of patients in the treatment group were more likely to be in the frontal lobe (40.4% vs. 28.6%), which may explain the higher rate of tumor resections in the SMA region in this group (22.9% vs. 4.8%). Forty-eight patients (58.5% of patients with data) had preserved MEPs postoperatively in the treatment group and 7 patients (100% of patients with information) in the sham group.

### Outcome measures: complete case analysis (sensitivity analysis)

**Fig. 1:**
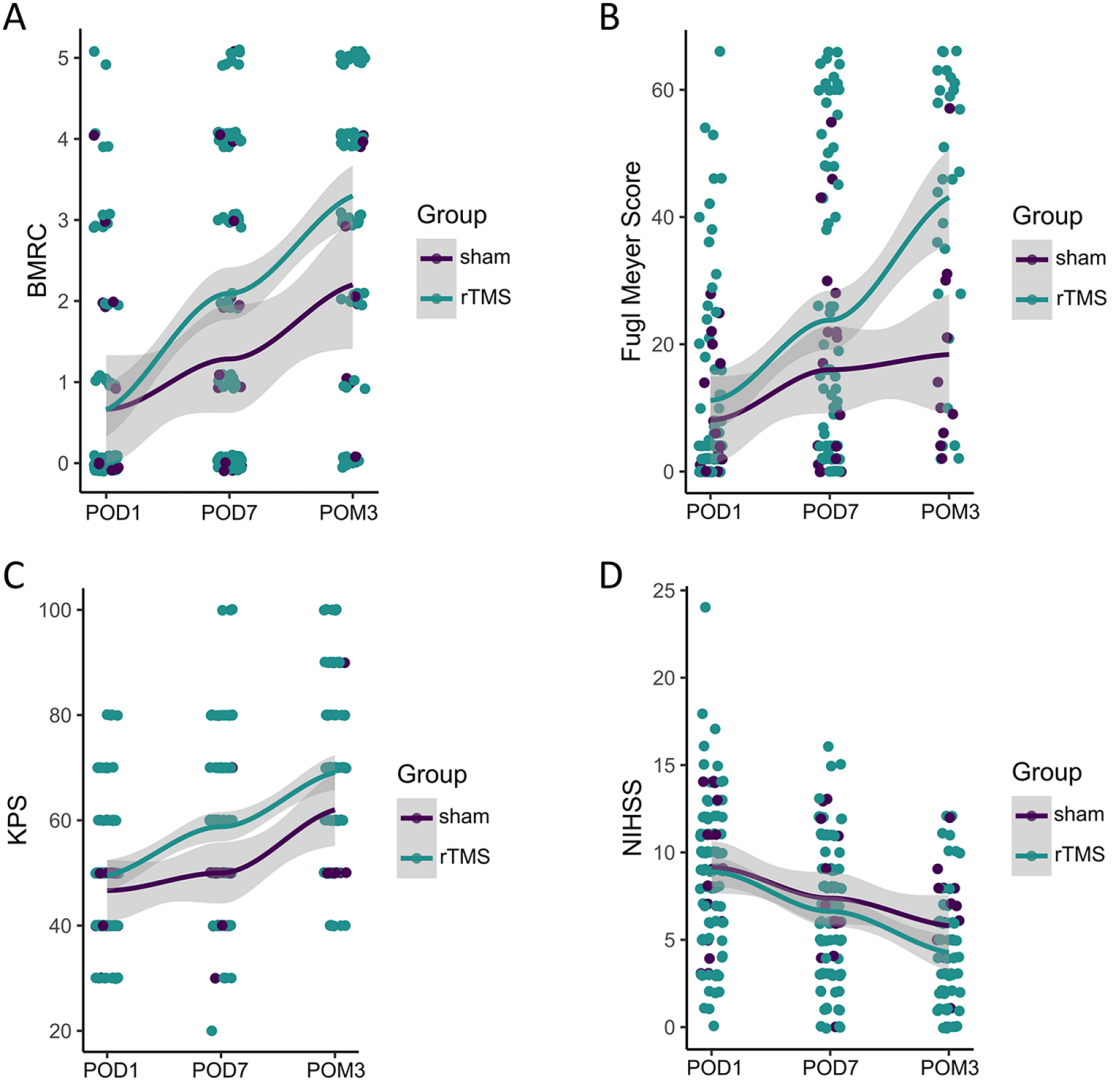
Scatter plot showing the outcome parameters by time point and group. All recorded values (without imputations) are shown for each time point, separated by groups (purple = Sham, blue = rTMS) for the BMRC (**a**, POD1: rTMS *n* = 90, sham *n* = 21; POD7: rTMS *n* = 96, sham *n* = 21; POM3: rTMS *n* = 71, sham *n* = 15), FMA (**b**, POD1: rTMS *n* = 73, sham *n* = 19; POD7: rTMS *n* = 75, sham *n* = 19; POM3: rTMS *n* = 29, sham *n* = 10), KPS (**c**, POD1: rTMS *n* = 96, sham *n* = 21; POD7: rTMS *n* = 106, sham *n* = 21; POM3: rTMS *n* = 78, sham *n* = 15) and the NIHSS (**d**, POD1: rTMS *n* = 85, sham *n* = 21; POD7: rTMS *n* = 83, sham *n* = 21; POM3: rTMS *n* = 57, sham *n* = 15). Colored dots represent values for individual subjects. Colored lines correspond to estimated group averages using locally estimated scatterplot smoothing (Loess) with the respective 95% confidence intervals

No patient experienced adverse events related to the intervention. The distribution of the outcome parameters by time points and the assigned groups are shown in Fig. 1. Descriptive outcomes by center and timepoint are reported in Table 3.

**Table 3:** Outcomes by time point, center, and group

	Pre-intervention (postoperative)		7 days		3 months	
	rTMS stimulation	Sham stimulation	rTMS stimulation	Sham stimulation	rTMS stimulation	Sham stimulation
FMA, median (IQR)	*N* = 73 4 (2–16)	*N* = 19 4 (0–16)	*N* = 75 13 (4–48)	*N* = 19 9 (1–25)	*N* = 29 47 (28–61)	*N* = 10 12 (7–28)
I^RCT^	*N* = 14 19 (1–41)	*N* = 13 3 (0–14)	*N* = 15 40 (13–60)	*N* = 15 4 (0–22)	*N* = 8 59 (45–62)	*N* = 5 14 (10–31)
II^RCT^	*N* = 15 6 (4–14)	*N* = 6 5 (3–14)	*N* = 15 16 (10–54)	*N* = 6 15 (5–28)	*N* = 14 45 (30–61)	*N* = 5 9 (4–21)
IIa	*N* = 29 2 (2–2)	-	*N* = 29 4 (2–9)	-	*N* = 7 46 (19–60)	-
III	-	-	-	-	-	-
IV	*N* = 17 8 (4–25)	-	*N* = 16 31 (13–50)	-	-	-
**KPS**,** mean (SD)**	*N* = 96 **49 (13)**	*N* = 21 **47 (11)**	*N* = 106 **59 (15)**	*N* = 21 **50 (13)**	*N* = 78 **69 (16)**	*N* = 15 **62 (17)**
I^RCT^	*N* = 15 56 (19)	*N* = 15 46 (13)	*N* = 15 60 (20)	*N* = 15 51 (15)	*N* = 10 78 (18)	*N* = 10 68 (18)
II^RCT^	*N* = 15 49 (8)	*N* = 6 48 (4)	*N* = 15 58 (12)	*N* = 6 54 (11)	*N* = 14 67 (18)	*N* = 5 50 (0)
IIa	*N* = 40 45 (9)	-	*N* = 40 53 (11)	-	*N* = 26 60 (10)	-
III	*N* = 26 53 (13)	-	*N* = 26 66 (18)	-	*N* = 21 79 (16)	-
IV	-	-	*N* = 10 62 (10)	-	*N* = 7 67 (14)	
**NIHSS**,** mean (SD)**	*N* = 85 **8.9 (4.3)**	*N* = 21 **9.1 (3.5)**	*N* = 83 **6.6 (3.9)**	*N* = 21 **7.4 (3.5)**	*N* = 57 **4.3 (3.4)**	*N* = 15 **5.8 (3.2)**
I^RCT^	*N* = 15 8.7 (6.2)	*N* = 15 8.4 (3.7)	*N* = 14 5.1 (2.7)	*N* = 15 7.3 (3.9)	*N* = 10 2.8 (2.3)	*N* = 10 4.4 (2.7)
II^RCT^	*N* = 15 9.9 (3.1)	*N* = 6 11.0 (2.3)	*N* = 15 5.9 (2.8)	*N* = 6 7.7 (2.4)	*N* = 14 4.6 (2.9)	*N* = 5 8.6 (2.1)
IIa	*N* = 40 10.2 (3.2)	-	*N* = 40 8.7 (3.7)	-	*N* = 25 5.9 (3.4)	-
III	-	-	-	-	-	-
IV	*N* = 15 4.5 (2.6)	-	*N* = 14 4.5 (2.6)	-	*N* = 8 0.5 (0.8)	-
**BMRC**	*N* = 90	*N* = 21	*N* = 96	*N* = 21	*N* = 71	*N* = 15
**0**	**65 (72.2%)**	**15 (71.4%)**	**31 (32.3%)**	**10 (47.6%)**	**7 (9.9%)**	**3 (20.0%)**
**1–2**	**13 (14.4%)**	**4 (19.1%)**	**21 (21.9%)**	**7 (33.3%)**	**15 (21.1%)**	**6 (40.0%)**
**3–5**	**12 (13.3%)**	**2 (9.5%)**	**44 (45.8%)**	**4 (19.1%)**	**49 (69.0%)**	**6 (40.0%)**
I^RCT^ 0 1–2 3–5	*N* = 15 8 (53.3%) 1 (6.7%) 6 (40.0%)	*N* = 15 11 (73.3%) 2 (13.3%) 2 (13.3%)	*N* = 15 3 (20.0%) 2 (13.3%) 10 (66.7%)	*N* = 15 8 (53.3%) 5 (33.3%) 2 (13.3%)	*N* = 11 1 (9.1%) 2 (18.2%) 8 (72.7%)	*N* = 11 2 (18.2%) 5 (45.5%) 4 (36.4%)
II^RCT^ 0 1–2 3–5	*N* = 15 8 (53.3%) 3 (20.0%) 4 (26.7%)	*N* = 6 4 (66.7%) 2 (33.3%) -	*N* = 15 1 (6.7%) 3 (20.0%) 11 (73.3%)	*N* = 6 2 (33.3%) 2 (33.3%) 2 (33.3%)	*N* = 14 1 (7.1%) 2 (14.3%) 11 (78.6%)	*N* = 4 1 (225.0%) 1 (25.0%) 2 (50.0%)
IIa 0 1–2 3–5	*N* = 40 36 (90.0%) 4 (10.0%) -	-	*N* = 40 21 (53.0%) 13 (33.0%) 6 (15.0%)	-	*N* = 25 5 (20.0%) 8 (32.0%) 12 (48.0%)	-
III 0 1–2 3–5	*N* = 20 13 (65.0%) 5 (25.0%) 2 (10.0%)	-	*N* = 26 6 (23.1%) 3 (11.5%) 17 (65.4%)	-	*N* = 21 - 3 (14.3%) 18 (85.7%)	-
IV 0 1–2 3–5	-	-	-	-	-	-

The table shows the outcomes per center and group of all 4 centers (I-IV). C (**I**), TU (**II and IIa**), L (**III**), MDA (**IV**). RCT = Randomized Controlled Trial

BMRC assessment was available in 111 patients (82.2%) before therapy, in 117 patients (86.7%) after therapy and in 86 patients after 3 months (63.7%). Patients in the treatment group had a higher probability of a better postoperative BMRC score immediately after treatment (estimated odds ratio for treatment vs. sham: 3.28, 95%CI: 1.08–9.99) and after 3 months (2.03, 95%CI: 0.65 to 6.39) compared to the sham group.

FMA score was available in 92 patients (68.1%) before therapy, in 94 patients (69.6%) after therapy, and in 39 patients after 3 months (28.9%). Patients in the treatment group had a similar postoperative FMA score immediately after treatment (estimated mean difference on log-transformed data treatment vs. sham: 0.28, 95% CI: -0.34–0.90) and after 3 months (0.14, 95% CI: -0.52 to 0.81) compared to patients in the sham group.

KPS score was available in 117 patients (86.7%) before therapy, in 127 patients (94.1%) after therapy, and in 93 patients after 3 months (68.9%). Patients in the treatment group had a substantially higher postoperative KPS score immediately after treatment (estimated mean difference treatment vs. sham: 11, 95% CI: 2 to 19) and after 3 months (11, 95% CI: 2–20) compared to patients in the sham group.

NIHSS assessment was obtained in 106 patients (78.5%) before therapy, in 104 patients (77.0%) after therapy, and in 72 patients (53.3%) after 3 months. Patients in the treatment group had similar NIHSS scores after 7 days (estimated mean difference treatment vs. sham: -1, 95%CI: -4 to 1) and a lower NIHSS score after 3 months (-3, 95%CI: -6 to -1) compared to patients in the sham group.

### Outcome measures: analysis of full data set after imputation of missing values

**Fig. 2:**
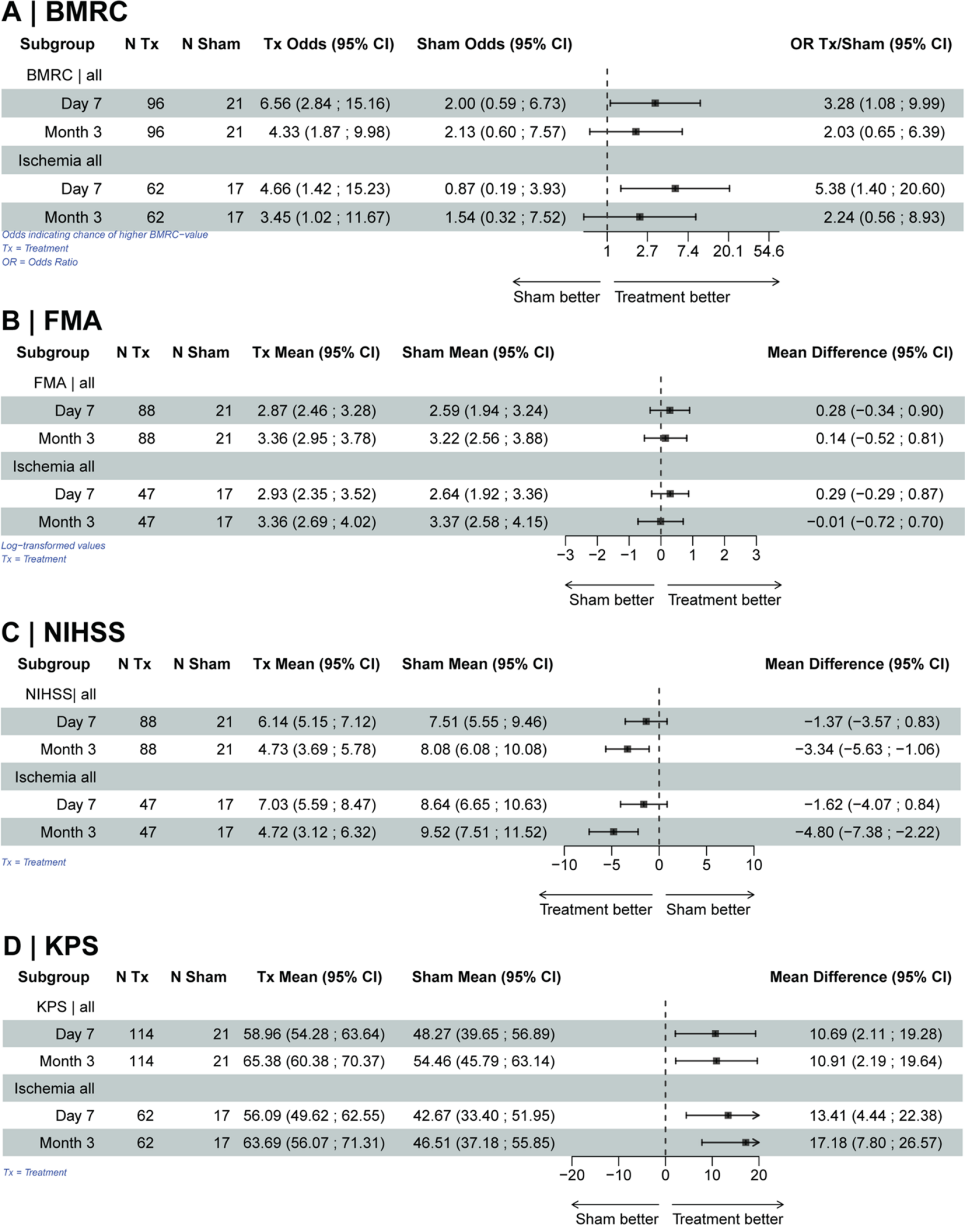
Forest plots based on imputed data showing the respective treatment effects. Treatment effects for the entire study population and for subgroups (patients with ischemia, patients with preserved postoperative MEPs) are shown. Estimates are based on multiple imputed datasets (30 complete datasets). **a**: Analysis of the BMRC based on ordinal logistic mixed models (random intercept models, random intercepts for patients) adjusted for the BMRC values before intervention, presence of SMA tumor, tumor histology and tumor location, time point, interaction of time point and treatment allocation, center and interaction of center and treatment allocation (OR: odds ratio). **b**: Analysis of the FMA based on mixed models (random intercept models, random intercepts for patients) with log-transformed FMA values, adjusted for log-transformed FMA values before intervention, presence of SMA tumor, tumor histology and tumor location, time point, interaction of time point and treatment allocation, center and interaction of center and treatment allocation. **c**: Analysis of NIHSS based on mixed models (random intercept models, random intercepts for patients) adjusted for NIHSS values before intervention, presence of SMA tumor, tumor histology and tumor location, time point, interaction of time point and treatment allocation, center and interaction of center and treatment allocation. **d**: Analysis of the KPS based on mixed models (random intercept models, random intercepts for patients) adjusted for KPS values before intervention, presence of SMA tumor, tumor histology and tumor location, time point, interaction of time point and treatment allocation, center and interaction of center and treatment allocation

The analysis of the treatment effects based on the imputed data is shown in Fig. 2 for the outcome parameters (BMRC, FMA, NIHSS, KPS) considering the subgroups (patients with ischemia / patients with preserved postoperative MEPs).

Similar to the sensitivity analysis, we observed a relevant treatment effect for the BMRC score on day 7 (after nrTMS treatment), which was even more pronounced in the subgroup of patients with proven ischemia (Fig. 2). Results were similar but not statistically robust for the long-term outcome after 3 months.

Analysis of the FMA showed a small advantage for patients in the rnTMS treatment group. The subgroup analysis revealed no substantial differences.

Patients in the nrTMS treatment group showed a lower/better NIHSS for long-term outcomes at 3 months, especially in the subgroup of patients with proven ischemia (Fig. 2). For the short-term outcome at day 7 (after treatment), the advantage of rnTMS treatment compared to sham stimulation was smaller.

When analyzing the KPS, a treatment benefit was observed at both time points, day 7 and long-term after 3 months (Fig. 2), again with a larger effect in the subgroup of patients with proven ischemia.

In the subgroup analysis investigating the presence of a center-specific impact on the treatment outcome, no discernible center-specific effect could be identified (Suppl. 1–4). Furthermore, there was no substantial difference in the subgroup of gliomas or in the comparison of primary tumors vs. recurrences (Suppl. 5, 6).

In 10 (43.5%) out of the 23 patients with a tumor in the SMA region, motor-eloquent ischemia was detected on the postoperative MRI. The treatment effect remained even when the patients with tumors in the SMA region were excluded from the analysis (Suppl. 7). However, the rnTMS treatment effect is somewhat more pronounced in the group of patients with SMA tumors.

Since only 53 patients (56.4%) in the treatment group received radiotherapy compared to the sham group with 16 patients (80%), we performed a subgroup analysis only with patients with adjuvant radiotherapy, which revealed no substantial difference (Suppl. 8).

### Number needed to treat

To observe an improvement in the BMRC score by at least one grade at 3 months, the NNT is 4 patients (95%CI: 2 to 40) and 4 patients (CI: 2–25) for the subgroup of proven ischemia. For KPS improvement of at least one score point after 3 months, the NNT is 3 patients (95%CI: 2 to 6) and 2 (CI: 2–3) for the subgroup of proven ischemia. No reliable NNTs can be calculated for FMA and NIHSS.

## Discussion

### Impact of nrTMS therapy for patients with acute paresis after tumor resection

This large international series of brain tumor patients with surgery-related paresis treated with contralateral nrTMS in patients with surgery-related paresis is the first multicenter analysis that showed the substantial benefit of this therapy in different healthcare systems. Contralateral nrTMS improved power (BMRC), general neurological status (NIHSS), functional oncological status (KPS) and fine motor skills (FMS) at 7 days and 3 months after surgery if combined with PT.

Microsurgical resection still is the initial therapeutic option for low-grade, high-grade, or recurrent glioma patients having the strongest oncological impact on the course of the disease to date [28, 29]. Due to modern technologies such as neuronavigation, preoperative diagnostics with nTMS and intraoperative neuromonitoring, injury to the motor system and postoperative paresis have become rare. However, its impact on the oncological course can be severe, causing reduced ambulation, independence and thus ineligibility for adjuvant treatment [26, 27]. In gliomas, the extent of resection is strongly correlated with survival. Conversely, if patients experience postoperatively a new neurological deficit, such as a paresis, this effect is negated, and patients have a poorer prognosis despite complete tumor resection [3]. Regaining functional status is therefore of great oncological importance. With this study showing even improvement in KPS, reveals the potential of reducing TCI by nrTMS to improve functional outcomes of new paresis after resection. Future studies may show to what extent recovery of other neurological deficits can be supported by non-invasive brain stimulation.

### Comparison to stroke patients

Enhancing motor recovery by rTMS has been studied extensively in stroke patients [4]. Despite the existence of numerous protocols and varying degrees of success, larger prospective studies are necessary to more accurately characterize the therapeutic effects and to better distinguish between responders and non-responders [16–18]. As in the present study, the down-regulation of the contralesional motor cortex by low-frequency rTMS suppressing TCI is the most frequently applied approach in stroke [30]. Although various low-frequency rTMS protocols (regarding stimulation intensity, number of sessions, etc.) exist in the literature, meta-analyses demonstrated the therapeutic effect of contralesional inhibitory low-frequency stimulation, which was found to be superior to ipsilesional high-frequency rTMS. Subgroup analyses from meta-analyses have shown that patients with subcortical strokes benefit more from rTMS than those with cortical strokes [13, 17]. The protocol used at our centers mainly originates from these stroke data. The pathophysiological mechanisms are similar when underperfused tissue—whether due to a stroke or perioperatively near the resection cavity—results in a loss of function that leads to subsequent motor deficits. Published data suggest a strengthening treatment effect when low-frequency nrTMS is used to reduce TCI as early as possible to counteract chronicity processes [31]. Patients who were treated earlier, within 2–3 months post-stroke, exhibited a stronger treatment effect compared to other included patients who were treated 3–12 months after the stroke. Some studies also reported successful rTMS stimulation administered directly after the stroke [31–33]. Consistently, we used low-frequency nrTMS to reduce TCI as early as possible after surgery.

Extrapolating the present results, we recommend a new analysis of preceding stroke trials aimed at reducing TCI by contralateral low-frequency nrTMS. Particularly minor subcortical strokes, which most closely resemble surgical ischemia and occur with delayed endovascular treatment, may be part of the subgroup of stroke patients who benefit the most from nrTMS.

Treatment effects were considerably better if subcortical ischemia was detected on postoperative MRI (Fig. 2). However, the percentage of patients with proven ischemia was significantly higher in the sham groups. Thus, the treatment effects could even have been even more pronounced if only patients with ischemia were enrolled. In patients with motor-eloquent ischemia, rTMS treatment may lead to better recovery compared to other patients (those with direct injury to the motor cortex or corticospinal tract), as the plasticity-inducing effects of rTMS (such as the recruitment of adjacent neuronal populations) may be more effectively induced in this group. However, this hypothesis should be further investigated in future research. We also performed subgroup analyses in patients with gliomas, in patients with tumors within the SMA region and a comparison between recurrent tumors and primary tumors. The rTMS treatment effect remained in all subgroups, however the treatment effect was somewhat more pronounced in the group of patients with SMA tumors, which can be explained by the recovery potential of the SMA syndrome. The observed treatment effect in the subgroup without tumors in the SMA region confirms that rTMS treatment is not based on the natural course of the SMA syndrome.

### Limitations

The major limitation of this study is the lack of randomization across all centers (and the associated uneven group distribution) and the slightly varying treatment setup (Table 1). The heterogeneity of the dataset is challenging; however all statistical methods were used to account for dataset heterogeneity and minimize potential analysis bias. After the publication of previous RCTs, the main goal of the current analysis was to showcase the applicability of the previous single-center randomized data across multiple centers, countries, and healthcare systems. Thus, this multicenter analysis offers real-world data that the treatment of surgery-related deficits using nrTMS is effective with a low NNT. It is worth noting that the stimulation protocols, durations, and integration with physical therapy varied slightly among the different centers (Table 1). Nonetheless, treatment effects remained strong. The proportion of patients with LGG was low in this study, so future studies with a larger sample size should investigate whether there is a different response to rTMS therapy based on different tumor entities. Based on the present analysis, no statement can be made regarding how tumor infiltration of the corpus callosum would have affected the rTMS treatment effect, as there were no corpus callosum infiltrating tumors present in the study population.

Some centers also did not exclude SMA tumors with consecutive SMA syndrome from the enrolment, which might hamper the results on the one hand since spontaneous improvement is frequent. On the other hand, glioma patients have a short window of opportunity concerning adjuvant treatment, which is only applied if functional independence is guaranteed. Restoring functional independence earlier than normally observed with spontaneous recovery thus provides an oncological rationale.

## Conclusion

This is the largest analysis of nrTMS therapy in patients suffering from acute functional deficits after brain tumor resection to date, with treatment replicated at 4 different centers in 3 countries. The multicenter analysis confirms the initial results of the (here co-analyzed) randomized-controlled trials evaluating the benefit of contralateral low-frequency nrTMS therapy in brain tumor patients with acute UE paresis after surgery. Furthermore, we confirm a considerably low NNT, especially if ischemia could be accounted for the neurological deterioration.

Therefore, contralateral nrTMS for transcallosal disinhibition can be offered to patients with surgery-related paralysis, especially if postoperative MRI showed ischemia in the motor system.

## Electronic supplementary material

Below is the link to the electronic supplementary material.

Supplementary Material 1

## Data Availability

No datasets were generated or analysed during the current study.

## Declarations

### Clinical trial registration/local institutional approval

Munich: NCT03982329, Local institutional registration: 12/15; Berlin: Deutsches Register für klinische Studien (DRKS00010043), Ethics Committee of the Charité (EA4/132/15); London: Local institutional registration 20230701NCR; Houston: NCT03606161.
