# Supplementary material for: Improving postsurgical paresis in brain tumor patients by transcranial magnetic stimulation
Source: J Neurooncol. 2025 Jan 23;172(2):417–28. doi: 10.1007/s11060-024-04931-9 (PMC11937064; doi:10.1007/s11060-024-04931-9)

**Supplementary Figure 1**

This figure shows treatment effects for the FMA in the RCT study population (excluding center II^a^,III and IV) in **A** and the subgroup of patients with postoperative motor-eloquent ischemia in **B** based on mixed models (random intercept models, random intercepts for patients) with log-transformed FMA values, adjusted for log-transformed FMA values before intervention, time point, interaction of time point and treatment allocation, centre, and interaction of centre and treatment allocation, presence of SMA tumor, tumor histology and tumor location, estimates are based on multiple imputed datasets (30 complete datasets).

| **1A** |  |
| --- | --- |
|  | 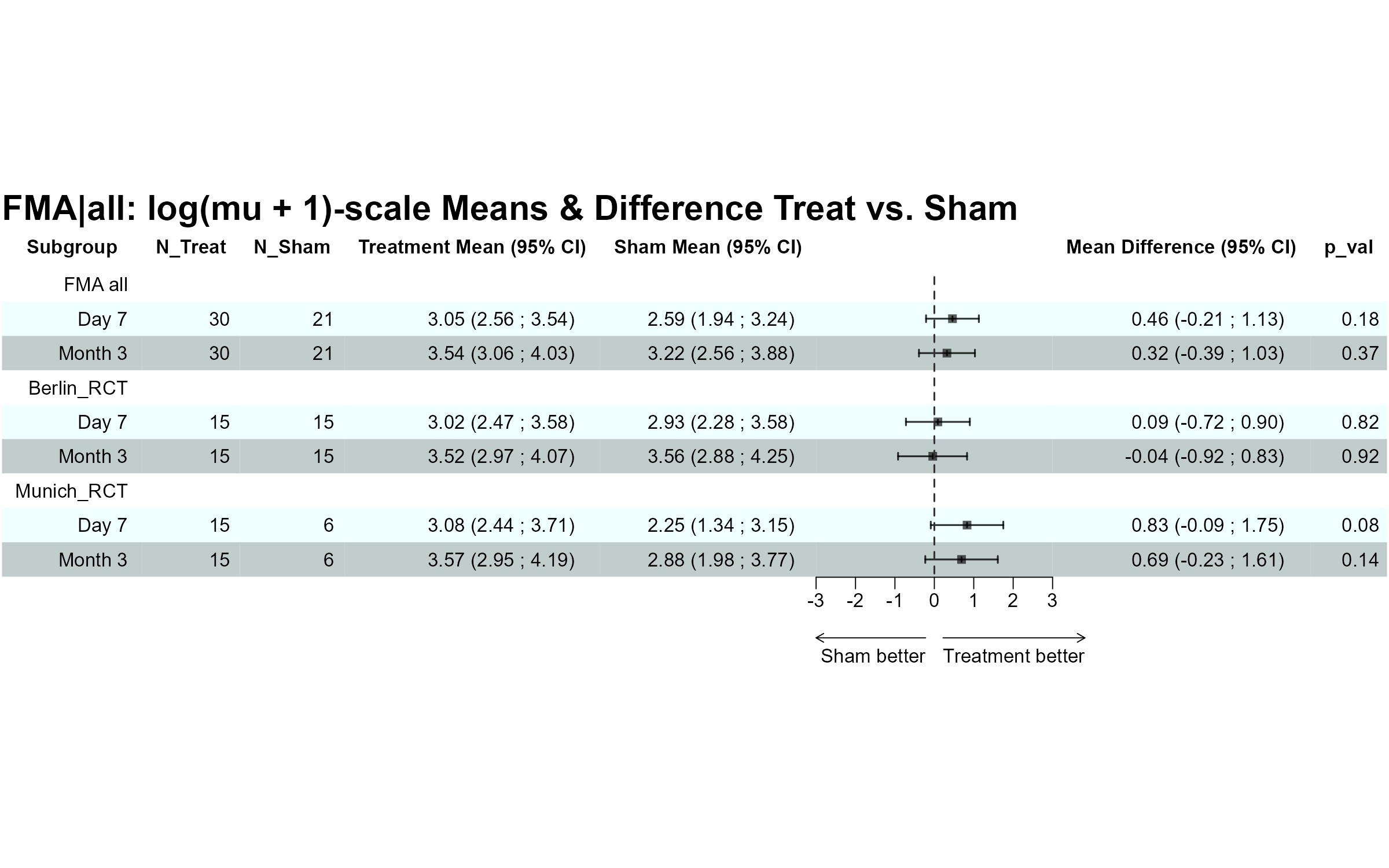 |
| **1B** |  |
|  | 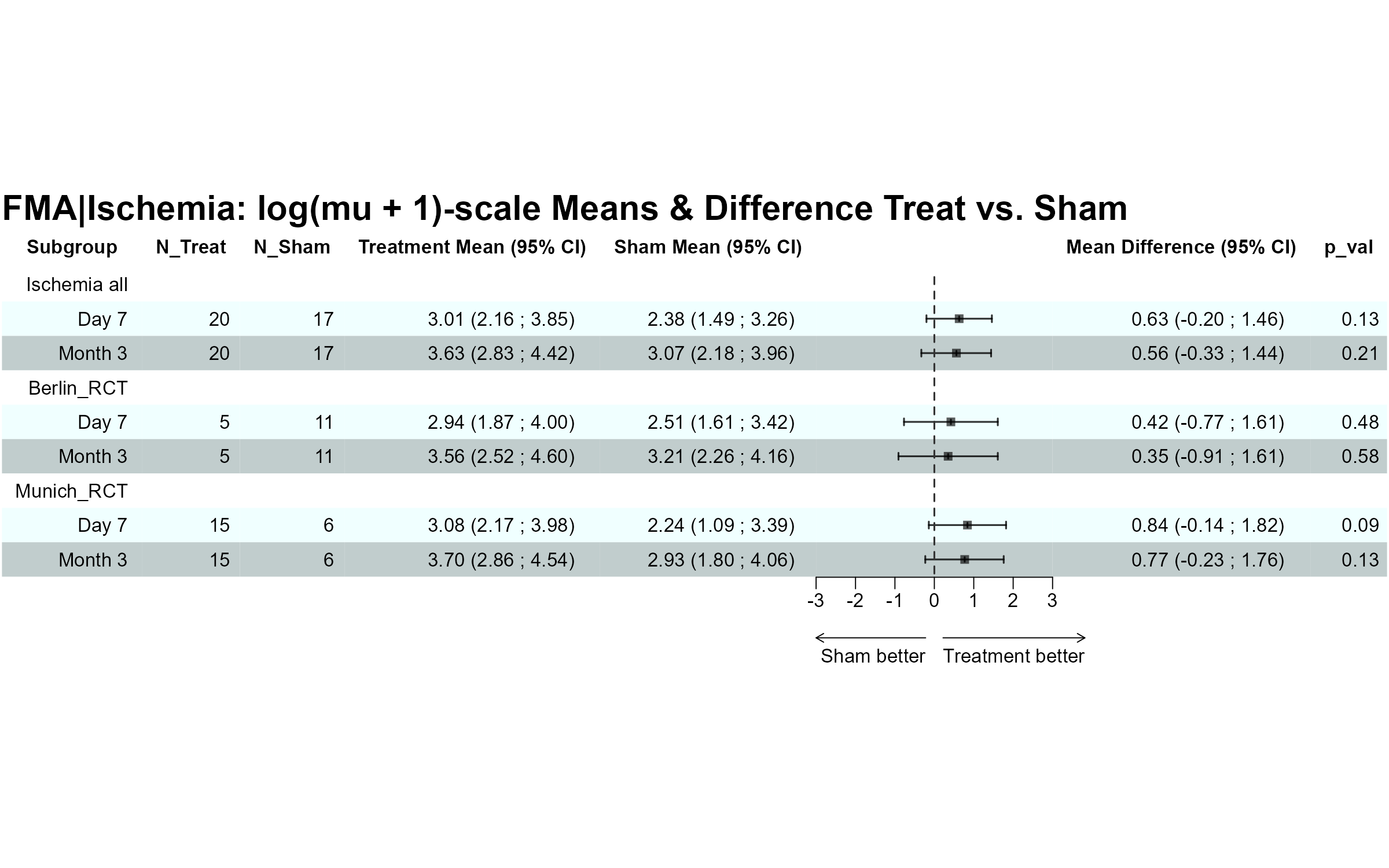 |

**Supplementary Figure 2**

This figure shows treatment effects for the NIHSS in the RCT study population (excluding center II^a^,III and IV) in **A** and the subgroup of patients with postoperative motor-eloquent ischemia in **B** based on mixed models (random intercept models, random intercepts for patients) adjusted for NIHSS values before intervention, time point, interaction of time point and treatment allocation, centre, and interaction of centre and treatment allocation, presence of SMA tumor, tumor histology and tumor location, estimates are based on multiple imputed datasets (30 complete datasets)

| **2A** |  |
| --- | --- |
|  | 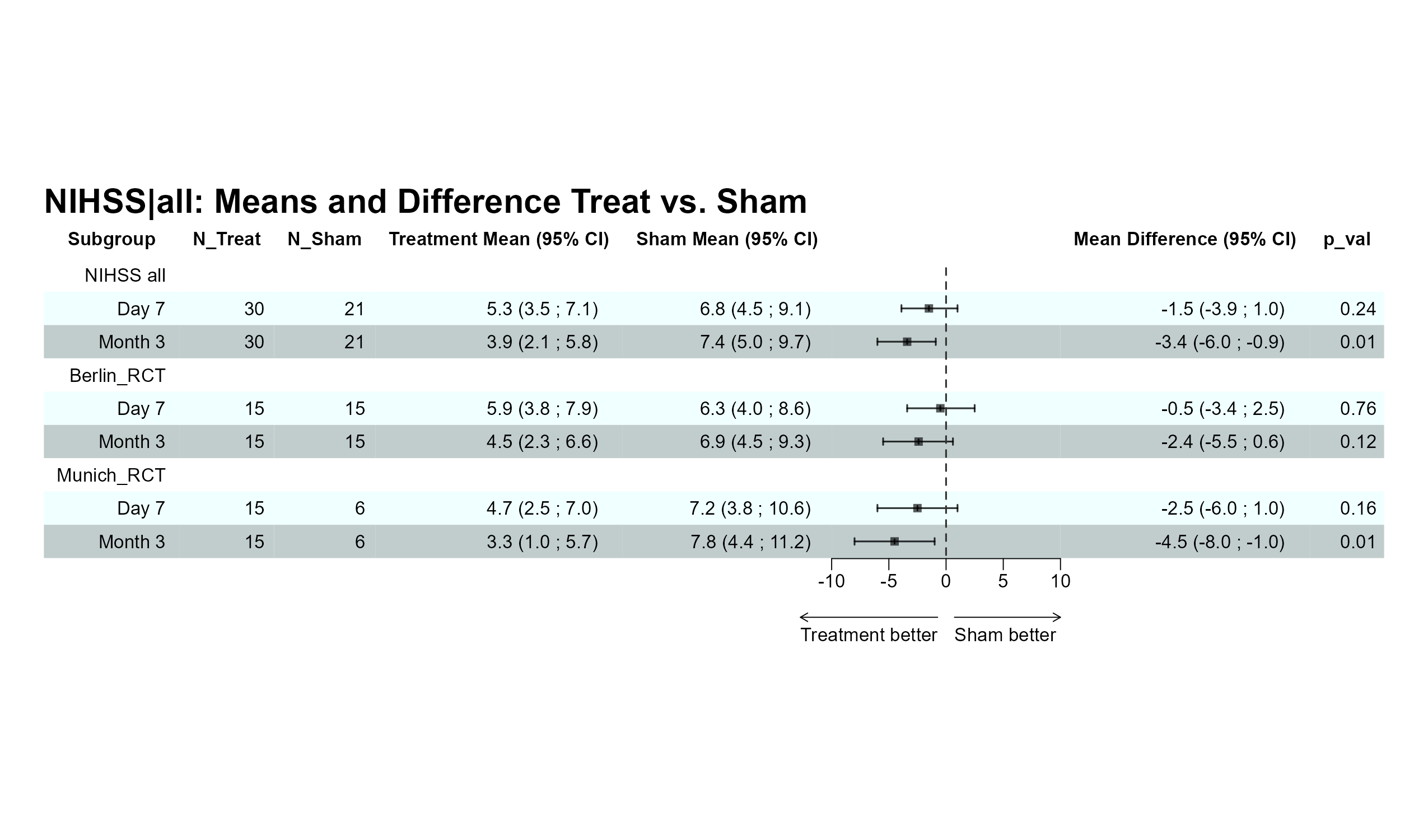 |
| **2B** |  |
|  | 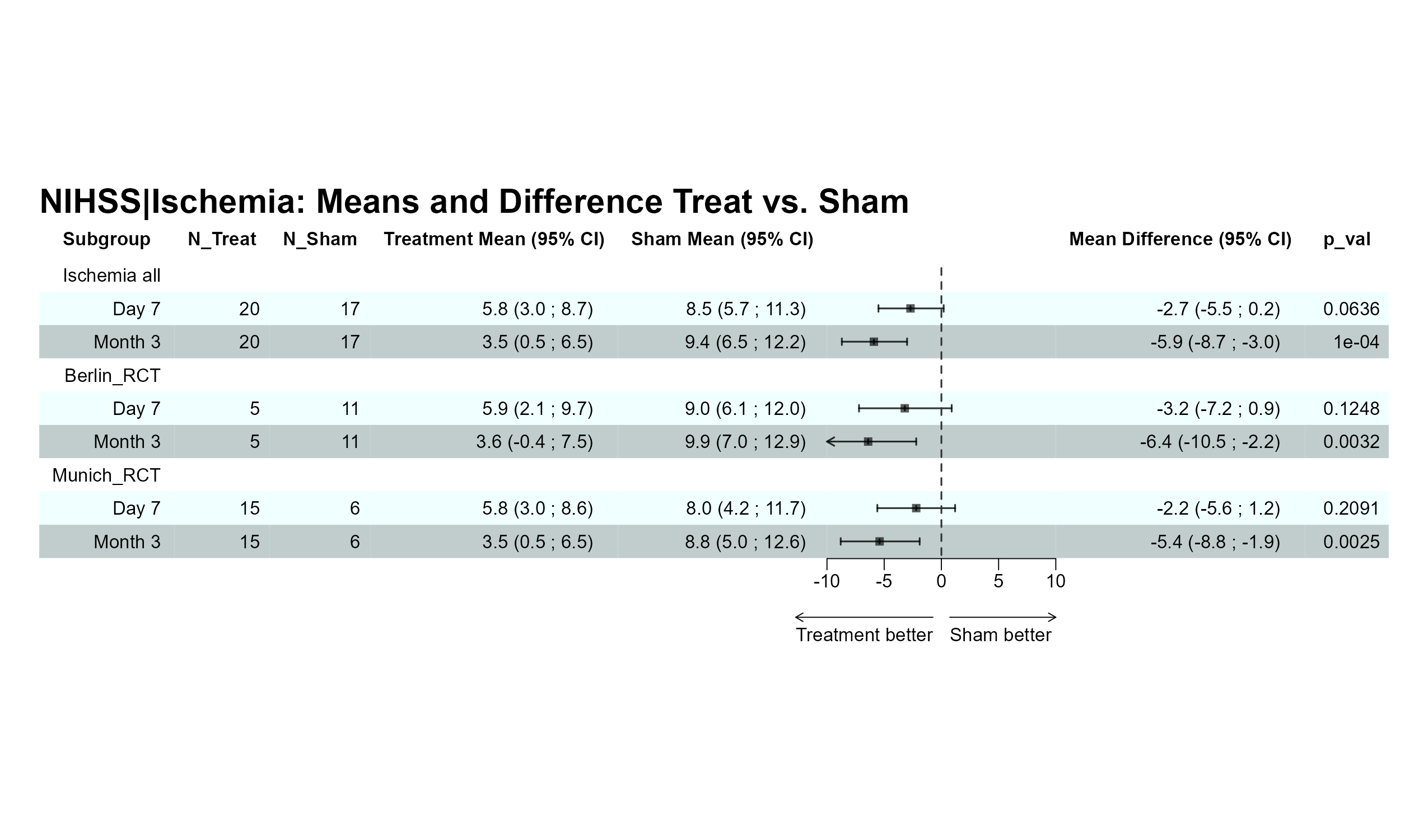 |

**Supplementary Figure 3**

This figure shows treatment effects for the KPS in the RCT study population (excluding center II^a^,III and IV) in **A** and the subgroup of patients with postoperative motor-eloquent ischemia in **B** based on mixed models (random intercept models, random intercepts for patients) adjusted for KPS values before intervention, time point, interaction of time point and treatment allocation, centre, and interaction of centre and treatment allocation, presence of SMA tumor, tumor histology and tumor location, estimates are based on multiple imputed datasets (30 complete datasets)

| **3A** |  |
| --- | --- |
|  | 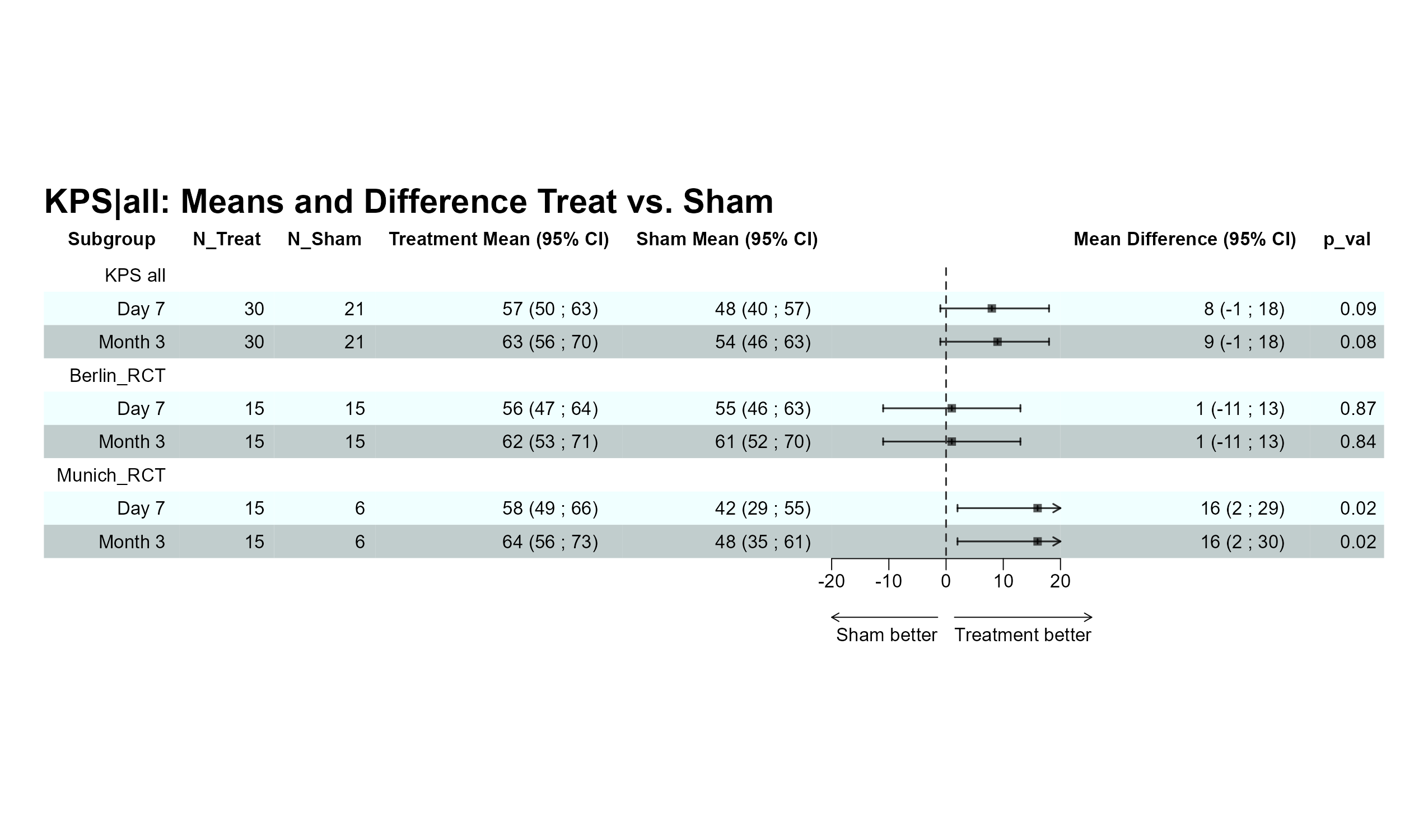 |
| **3B** |  |
|  | 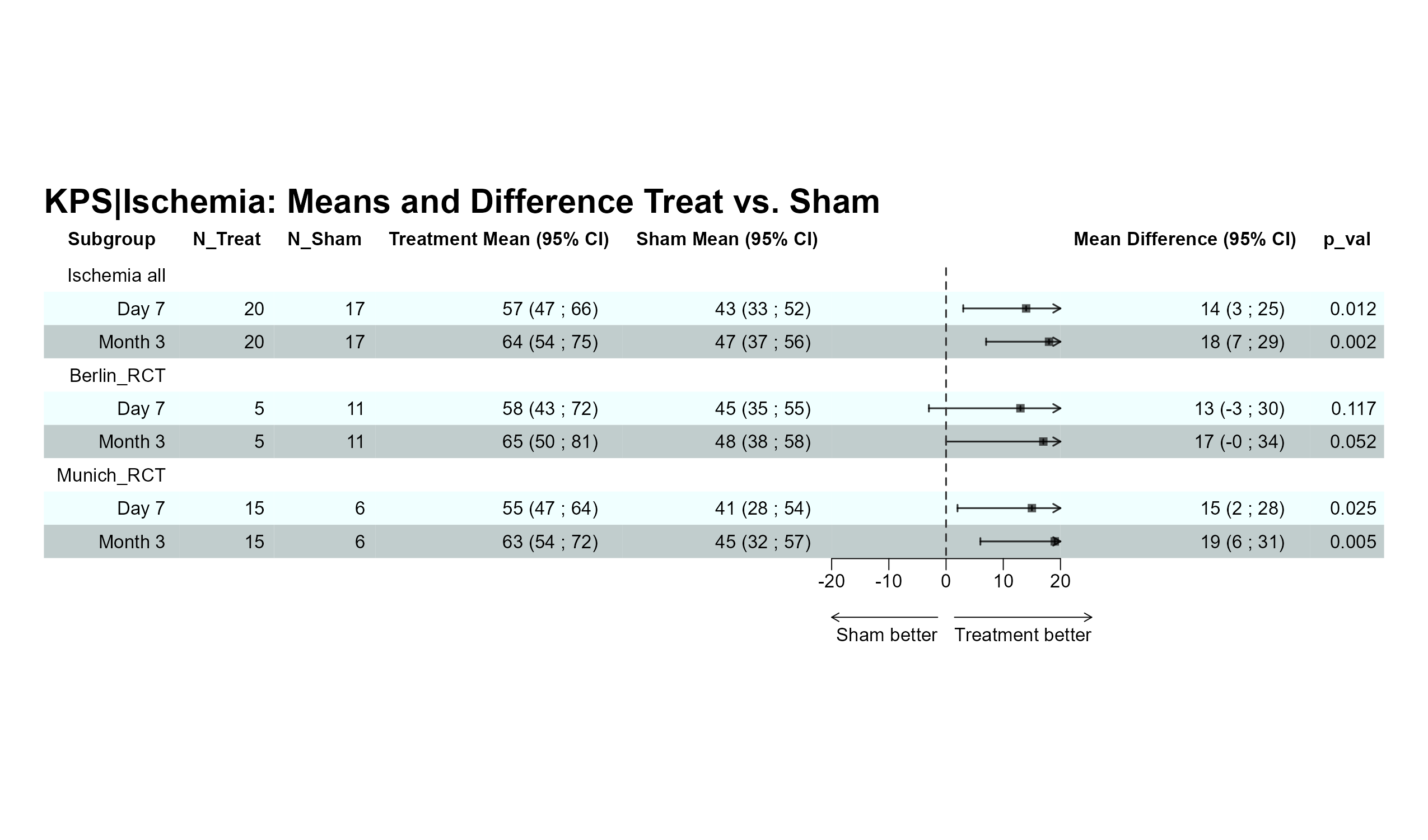 |

**Supplementary Figure 4**

This figure shows treatment effects for the BMRC in the RCT study population (excluding center II^a^,III and IV) in **A** and the subgroup of patients with postoperative motor-eloquent ischemia in **B** based on ordinal logistic mixed models (random intercept models, random intercepts for patients) adjusted for BMRC values before intervention, time point, interaction of time point and treatment allocation, centre, and interaction of centre and treatment allocation, presence of SMA tumor, tumor histology and tumor location, estimates are based on multiple imputed datasets (30 complete datasets) (OR: odds ratio)

| **4A** |  |
| --- | --- |
|  | 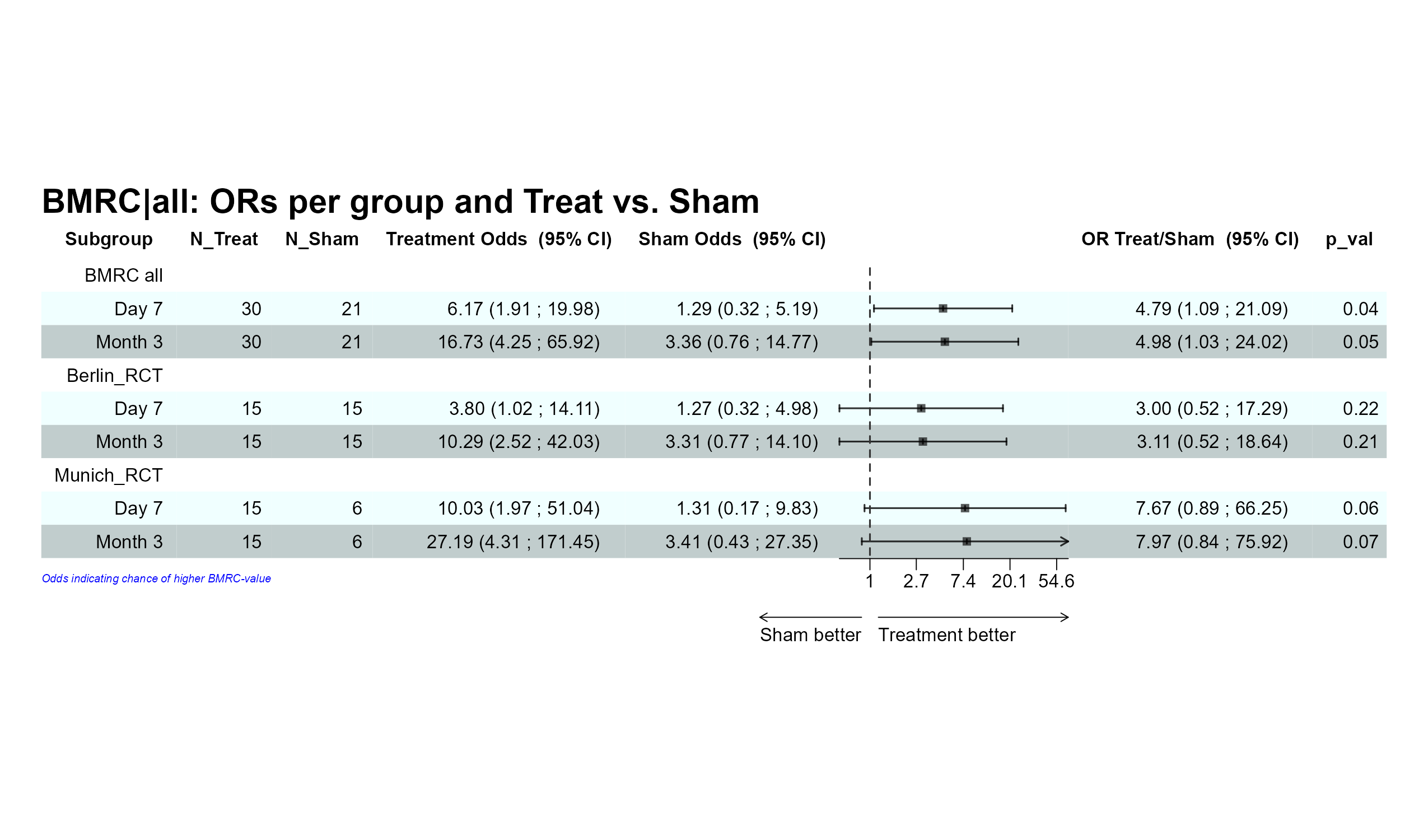 |
| **4B** |  |
|  | 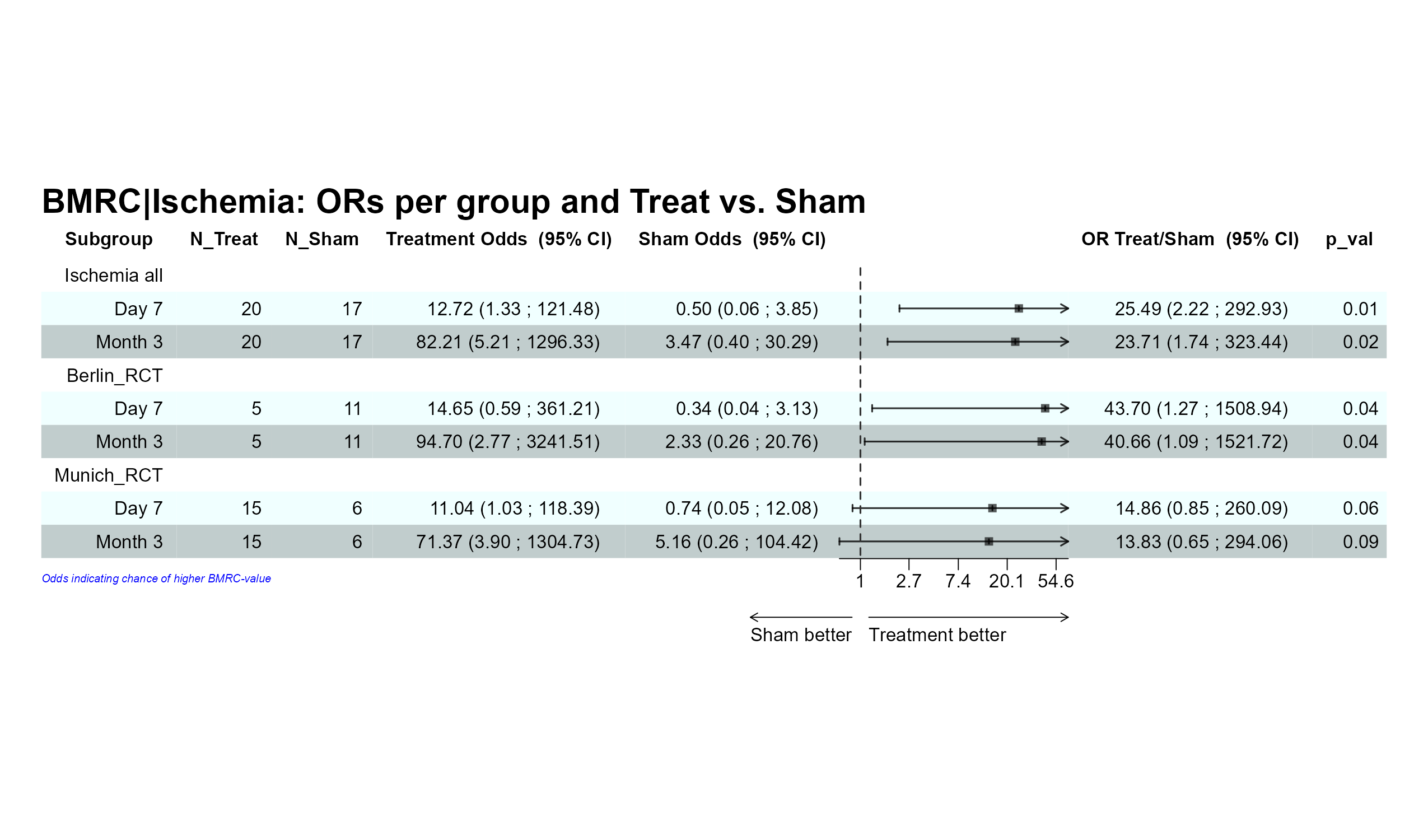 |

**Supplementary Figure 5**

Treatment effects for FMA, BMRC, NIHSS and KPS in study subgroup population with glioma tumor histology. Estimates are based on multiple imputed datasets (30 complete datasets) and mixed models as in the main analyses. (Note: Center Texas was excluded for BMRC outcome and Center London was excluded for FMA and NIHSS outcomes due to missing information in these outcomes)


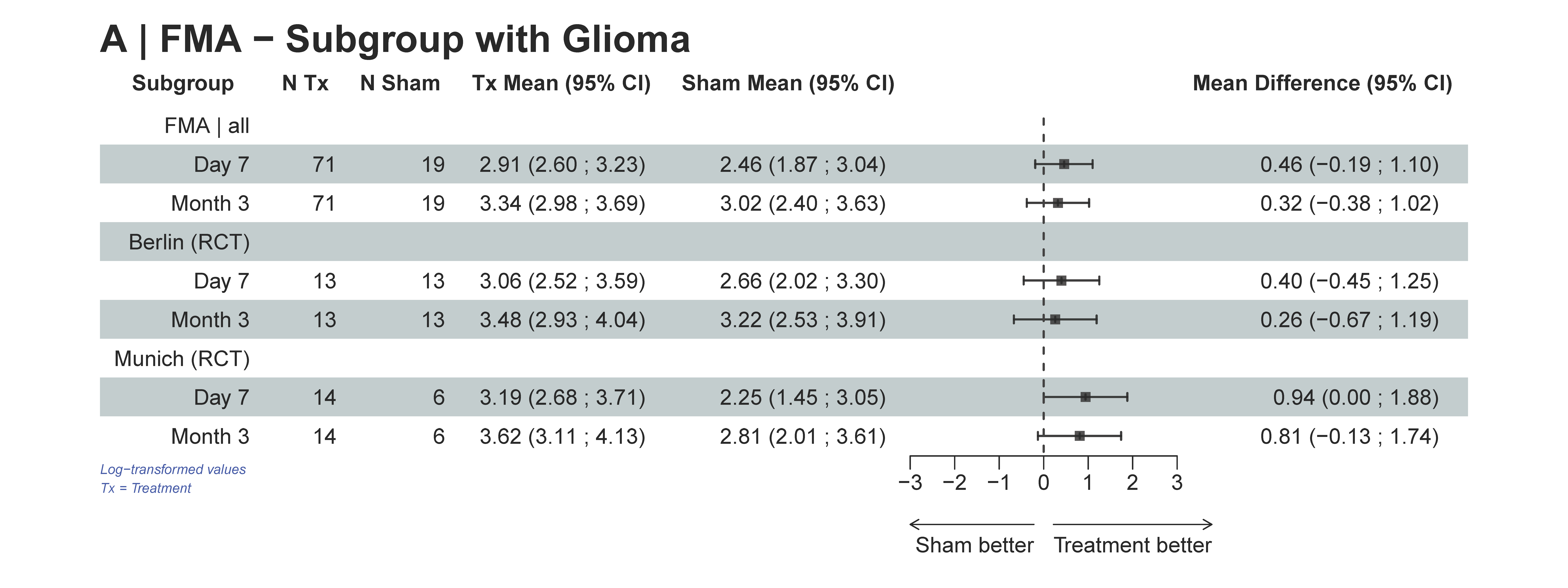


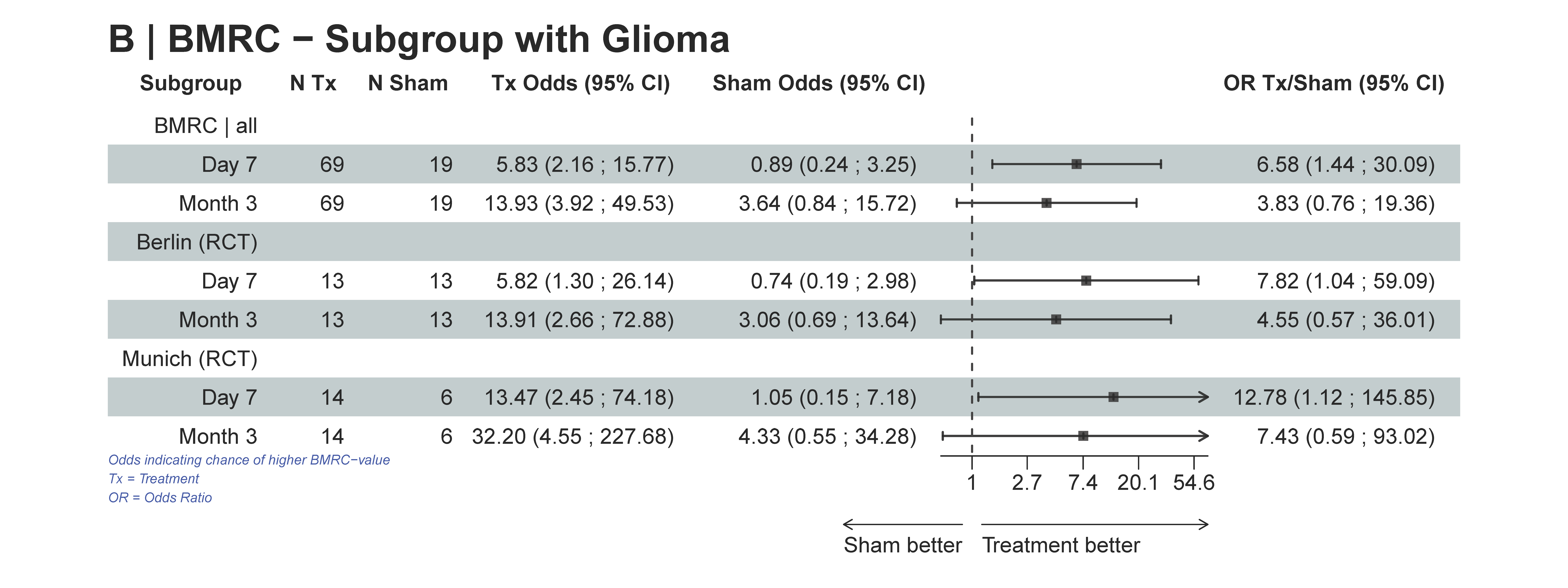


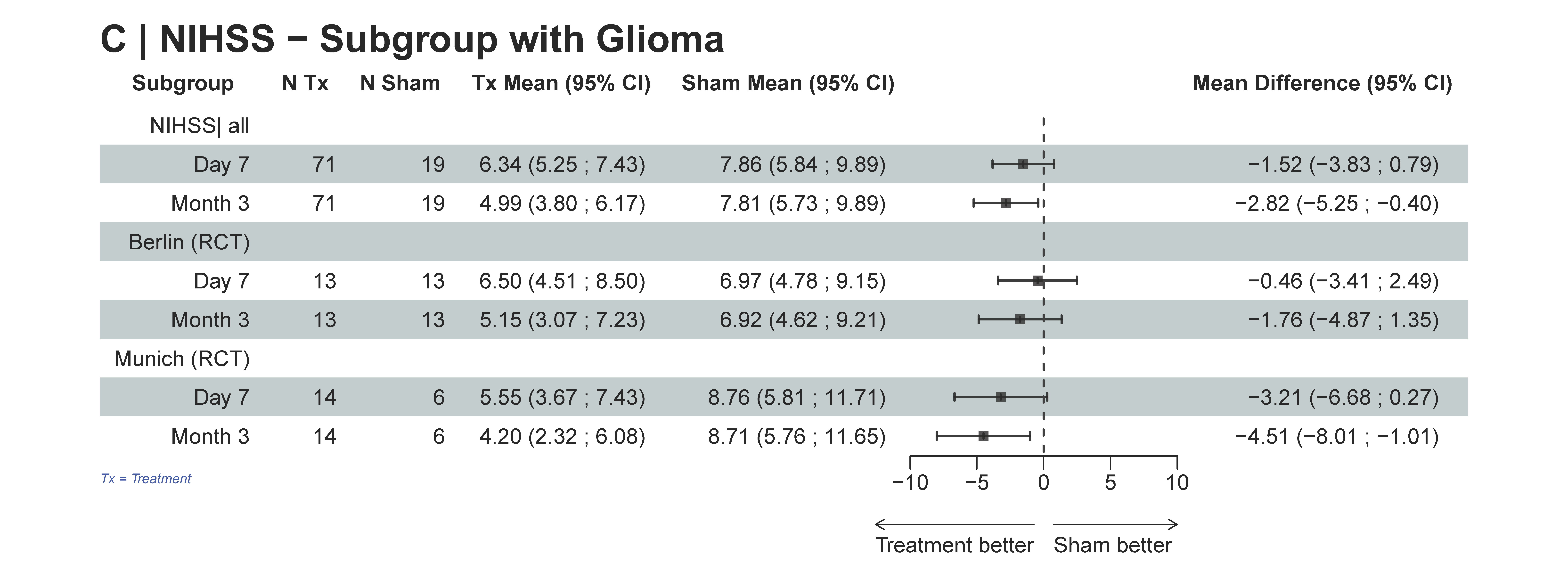


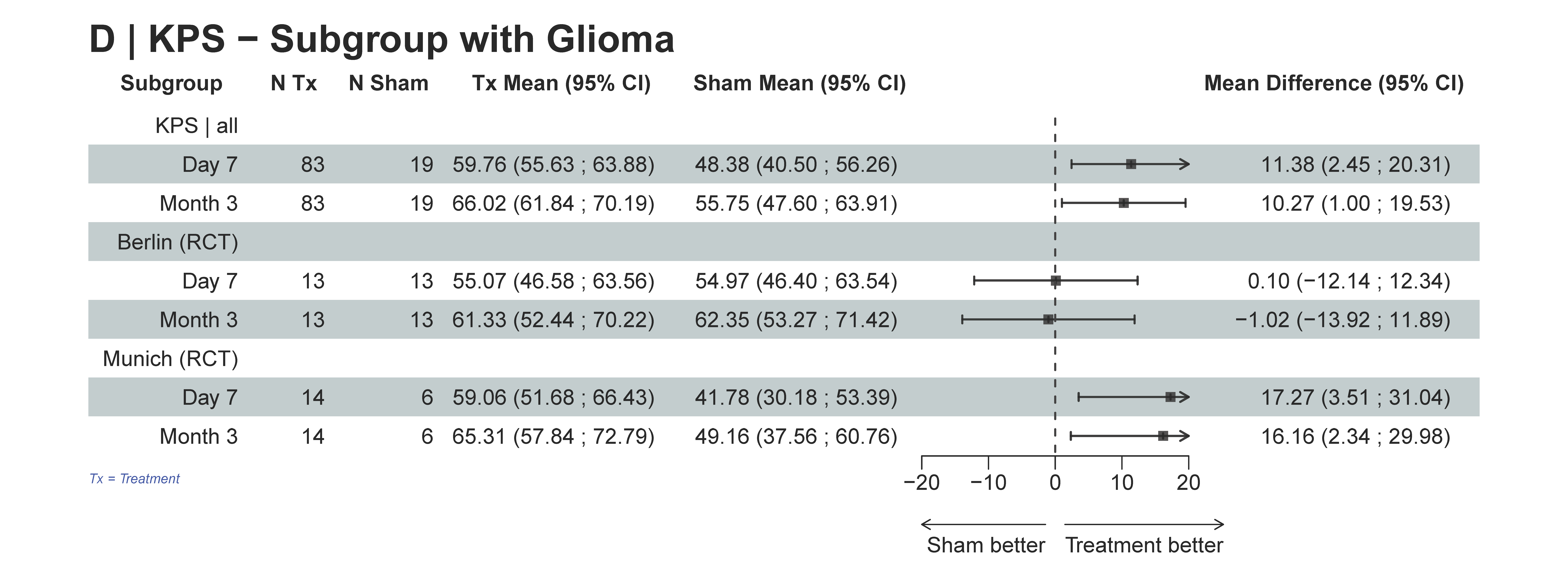


**Supplementary Figure 6**

Treatment effects for FMA, BMRC, NIHSS and KPS by recurrence state (recurrent tumor / no recurrent tumor). Estimates are based on multiple imputed datasets (30 complete datasets) and mixed models including interaction terms for treatment allocation and recurrence state, adjusted for Baseline and time point. For outcomes FMA, BMRC and KPS values greater than 0 indicate tendency of Treatment being better and for NIHSS the same holds true for values lesser than 0.





**Supplementary Figure 7**

Treatment effects for FMA, BMRC, NIHSS and KPS in study subgroup population (without tumor within the SMA region). The estimates are based on multiple imputed datasets (30 complete datasets) and mixed models including an interaction term for treatment allocation and recurrence state, adjusted for baseline and time point. (note: Centre in Texas was excluded due to missing information on recurrence state, Centre in London was excluded for analysis of FMA, and NIHSS due to missing information in outcomes)
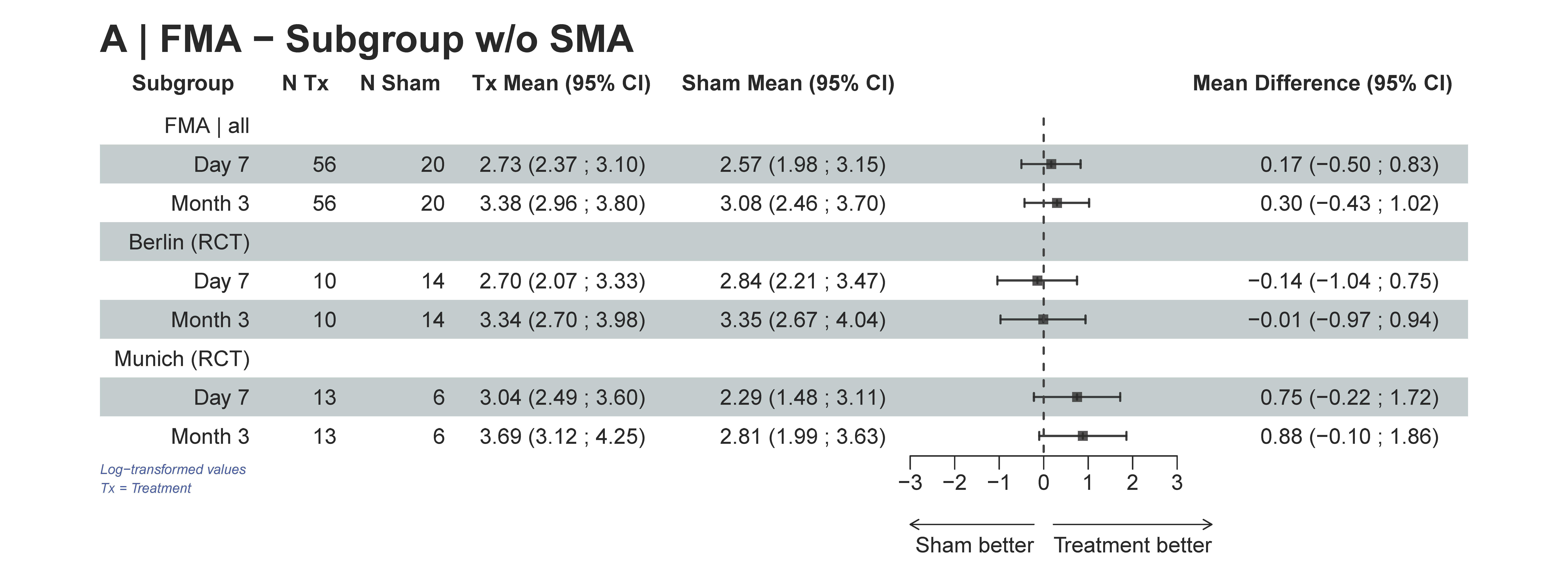


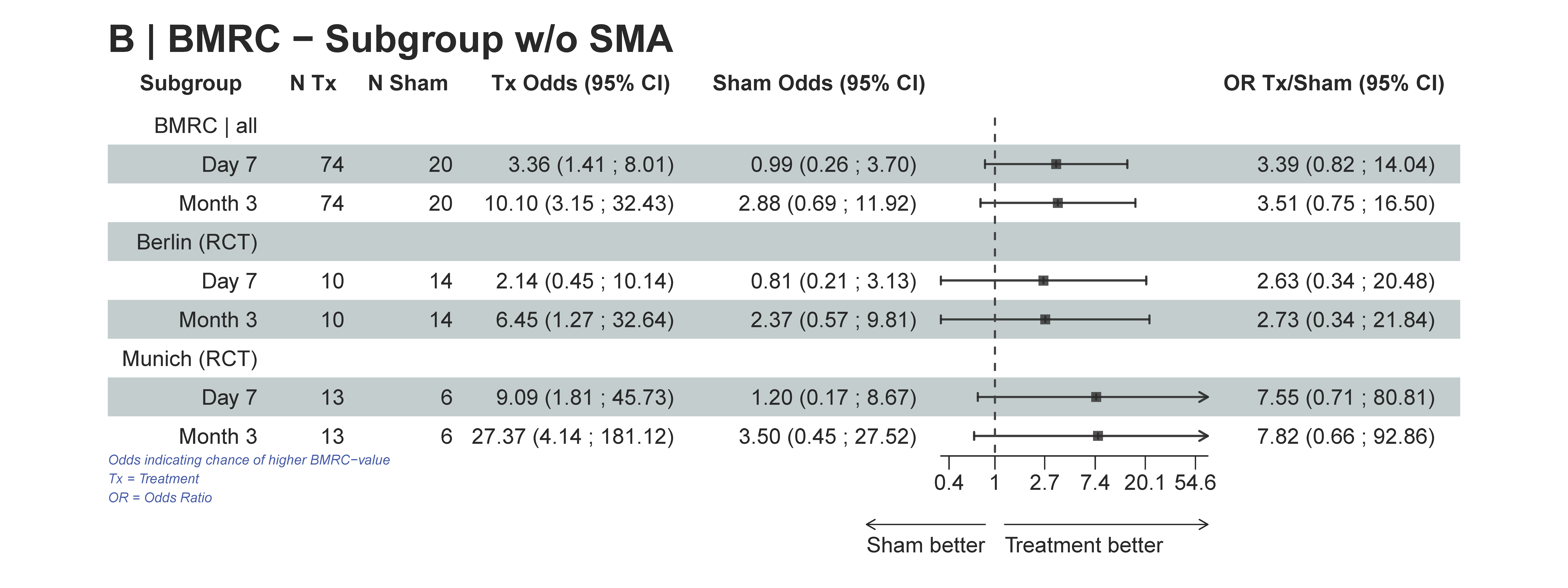


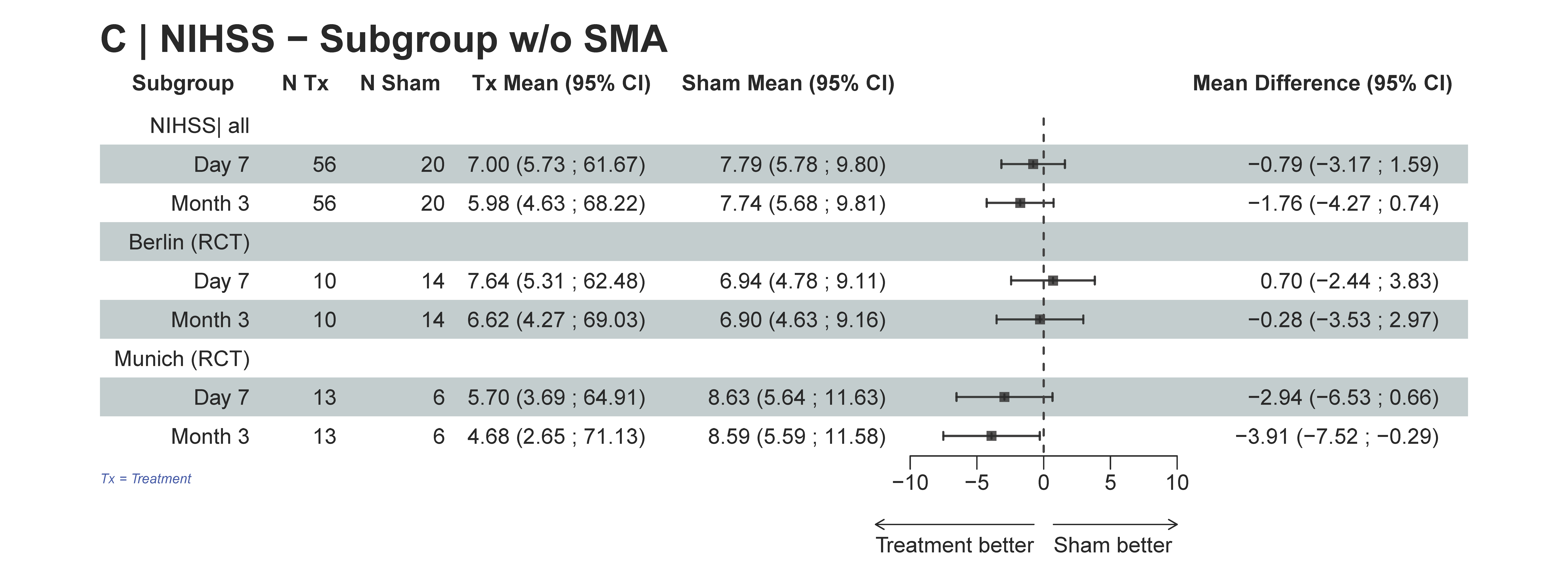


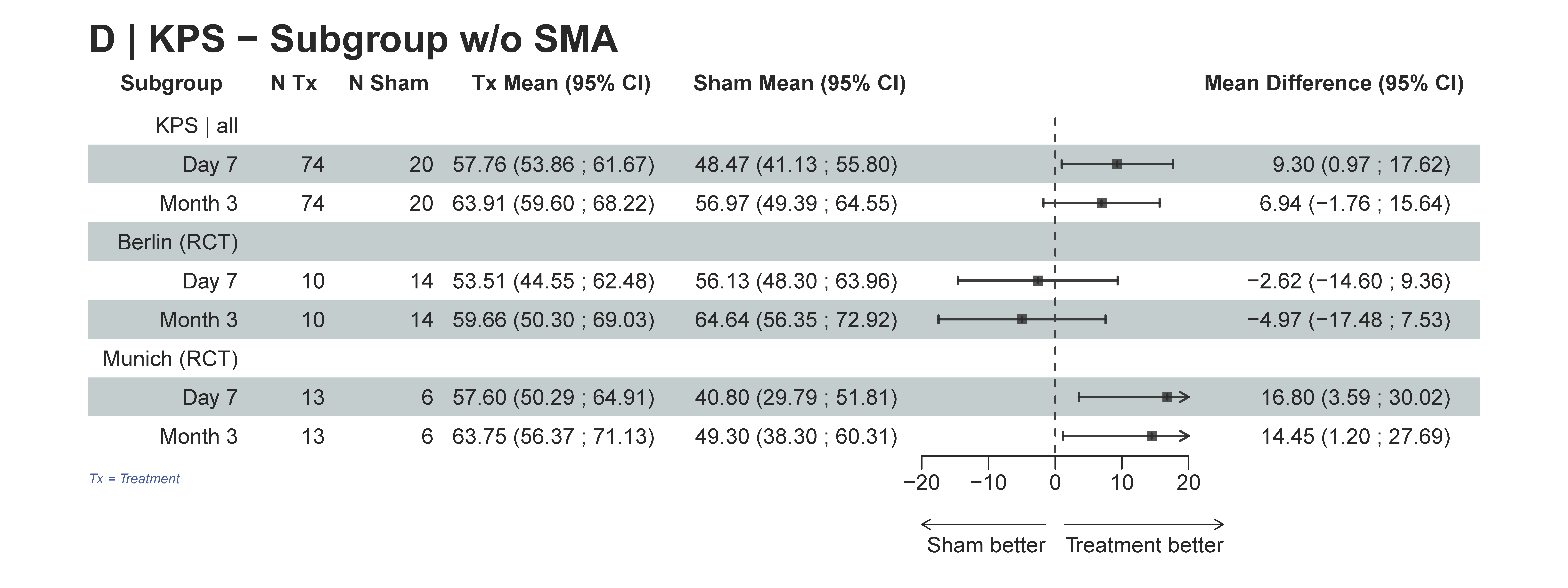


**Supplementary Figure 8**

Treatment effects for FMA, BMRC, NIHSS and KPS in study subgroup population (with adjuvant radiotherapy). Estimates are based on multiple imputed datasets (30 complete datasets) and mixed models as in the main analyses. (Note: Center Texas was excluded for all outcomes and Center London was excluded for FMA and NIHSS outcomes due to missing information in these outcomes)


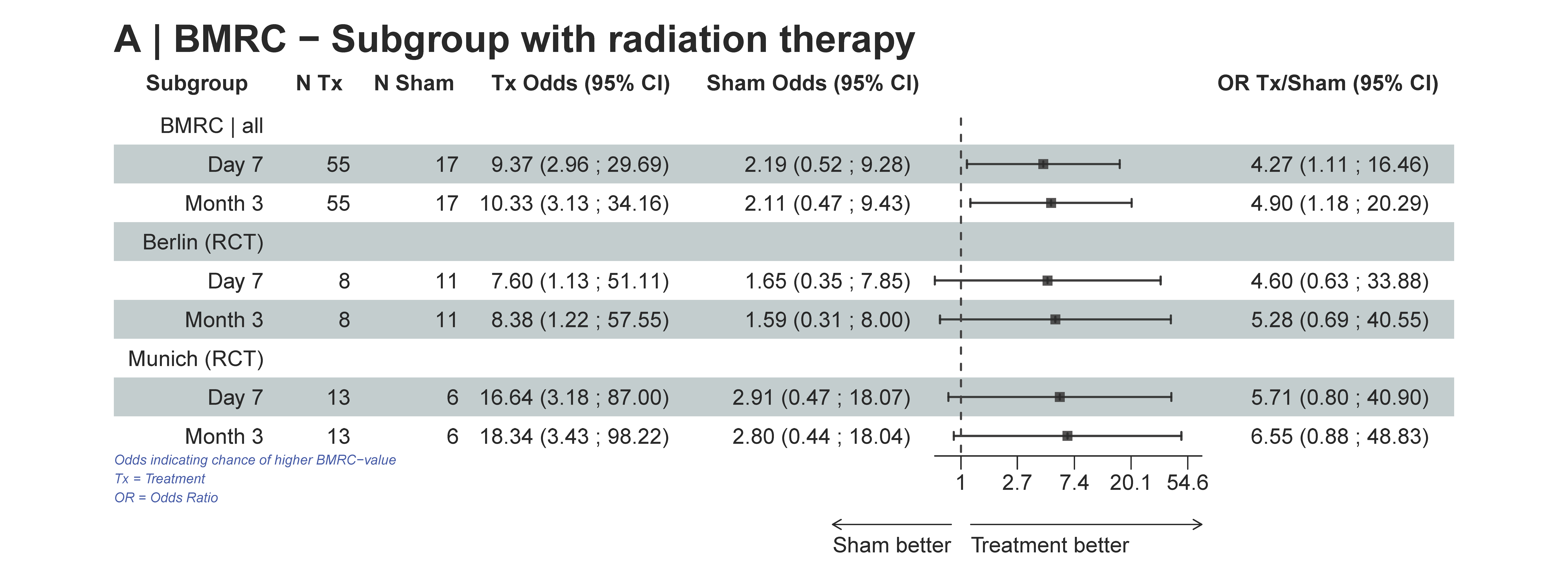

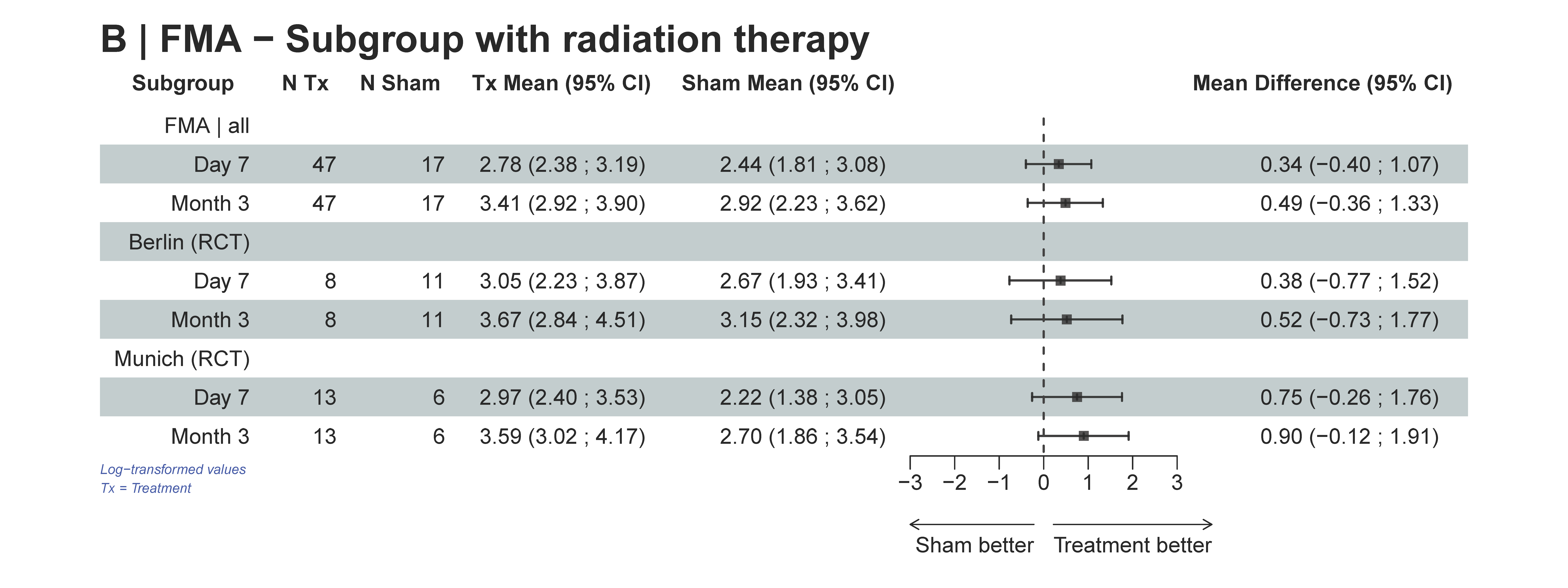

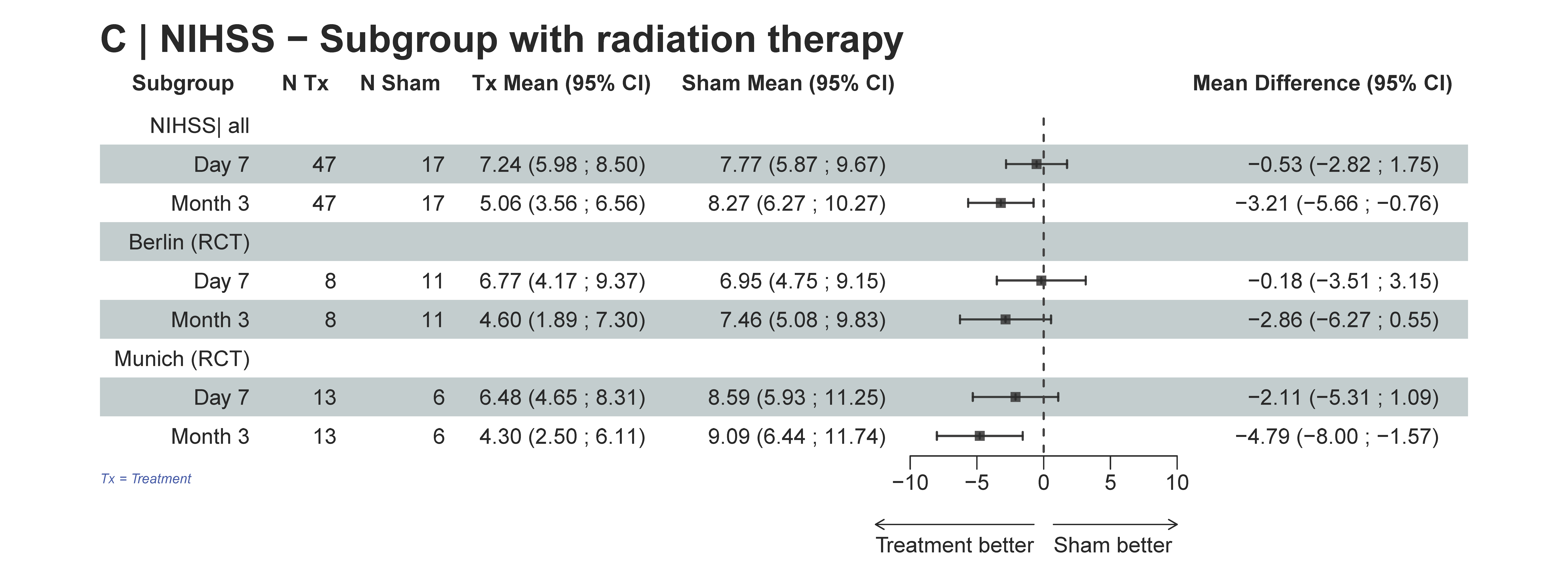

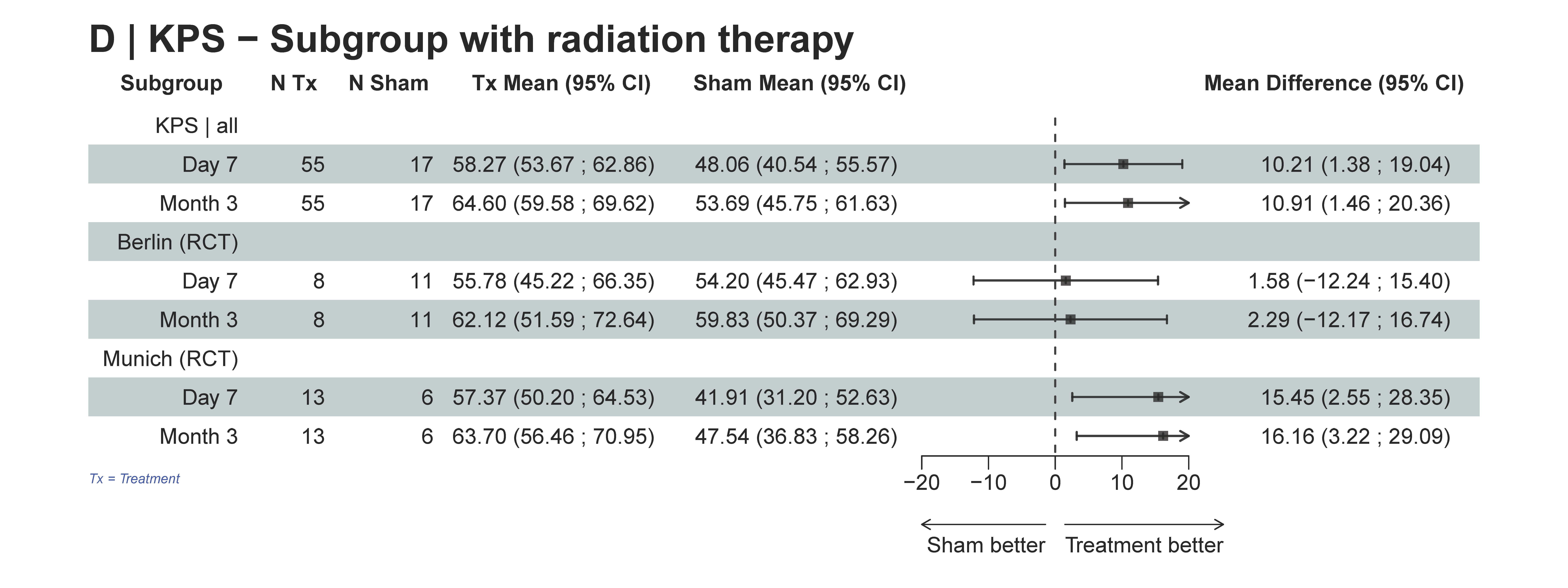

Supplement: Supplementary file 1 — Supplementary Material 1 [file 11060_2024_4931_MOESM1_ESM.docx]
